# Mate‐guarding success depends on male investment in a butterfly

**DOI:** 10.1002/ece3.10533

**Published:** 2023-09-17

**Authors:** Ádám Gór, Zsolt Lang, Kata Pásztor, Viktor Szigeti, Flóra Vajna, János Kis

**Affiliations:** ^1^ Doctoral School of Veterinary Science University of Veterinary Medicine Budapest Budapest Hungary; ^2^ Department of Biostatistics University of Veterinary Medicine Budapest Budapest Hungary; ^3^ Doctoral School of Biological Sciences Hungarian University of Agriculture and Life Sciences Gödöllő Hungary; ^4^ Lendület Ecosystem Services Research Group Institute of Ecology and Botany, Centre for Ecological Research, ELKH (Eötvös Lóránd Research Network) Vácrátót Hungary; ^5^ Department of Zoology University of Veterinary Medicine Budapest Budapest Hungary

**Keywords:** Copulatory opening APpendix, ditrysia, event history, mating system, multistate modelling, time‐series analysis

## Abstract

Males of many insects, including butterflies, produce mate‐guarding devices, such as mating plugs, to prolong guarding and prevent future female matings in the male's absence. In a few butterflies, large external mate‐guarding devices, that is, sphragides, occur. Gór et al. (*Behaviour*, 160, 2023 and 515−557) found conspicuously large size and morphological variation of mate‐guarding devices within a single population of the potentially polyandrous Clouded Apollo (*Parnassius mnemosyne*, L.) butterfly. They termed the externally visible male‐produced devices as Copulatory opening APpendices (CAP) consisting of small devices, termed small CAPs and the much larger shield (i.e. sphragis). Our aim was to reveal CAP replacement dynamics within females during their lifetime and to understand how male investment into small CAPs or shields was (i) related to CAP persistence on the female, that is securing paternity, (ii) associated with female quality, measured as size and (iii) with actual adult sex ratio. We investigated a univoltine Clouded Apollo population to estimate CAP replacement risks, using multistate survival models, in an extensive observational study through 6 years based on mark‐recapture. Shields were the most frequent mate‐guarding devices and were more persistent than small CAPs, often lasting for life, excluding future matings. Thus, most females bearing a shield were deprived of postcopulatory female choice, and the genetic variance in their offspring could be reduced compared to those bearing small CAPs, thus mating more often. The ratio of shields to all CAPs gradually decreased towards the end of the flight period. Males were more prone to produce a shield when mating females with wider thoraces and when the ratio of males (i.e. competition) was higher in the population. To our best knowledge, this is the first quantitative study to investigate potential factors on which male investment in mate‐guarding devices may depend, and how the variation in these devices impacts CAP persistence on females.

## Graphical abstract

This is the first quantitative study to investigate potential factors on which male butterfly investment in mate‐guarding devices may depend, and how the variation in these devices impacts their persistence on females. The study's aim was to reveal the alternative mate‐guarding devices' (i.e. CAP, Copulatory opening APpendix) replacement dynamics within Clouded Apollo (*Parnassius mnemosyne*) females during their lifetime and to understand how male investment into these devices is (i) related to their persistence on the female, that is, securing paternity, (ii) associated with female quality, measured as size and (iii) with actual adult sex ratio. The larger mate‐guarding devices (shields) are the most frequent ones and are more persistent than the smaller (small CAPs), often lasting for life, excluding future matings, thus, most females bearing a large mate‐guarding device are deprived of postcopulatory female choice, and the genetic variance in their offspring may be reduced.

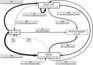

## 1 INTRODUCTION

Attributed to anisogamy, the evolutionary interest of most species' males is to mate as many females as possible, while females are interested in selecting the best partners to mate, and these induce intense male–male competition for mating (Chapman et al., 2003; Edward, 2015). Male competition may result in a reproductive load, that is, reduced fitness in populations (Holland & Rice, 1999; Okada et al., 2021). Polyandrous females' cryptic postcopulatory mate choice may mitigate one aspect of this load, the harmful impacts of potential male aggression driven by competition (Firman et al., 2017). In turn, males may guard their mates to prevent their future matings and secure paternity (Benvenuto & Weeks, 2012; Parker, 1970; Simmons, 2002). Male mate‐guarding has been found in a broad variety of taxa from rotifers (Schröder, 2003) to primates (Alberts et al., 1996).

Investment in guarding may depend on several factors. (i) The level of male–male competition increases with the increasing population male ratio (Weir et al., 2011), selecting for males to invest more in guarding (Jormalainen, 1998; Takeshita & Henmi, 2010). (ii) The amount of affordable resources and time to spend on guarding may depend on individual variation (Cueva Del Castillo, 2003), for example, condition among competitors. (iii) The expected reproductive value of the mating partner (Manning, 1975; Schöfl & Taborsky, 2002; Shuster, 1981; Thompson & Manning, 1981) may also depend on individual variation, for example, variance in female condition. The latter may result in males assessing female quality before mating. In most insects, larger female body size is associated with better quality (Gilbert, 1984; Honěk, 1993; Oberhauser, 1997; Okada et al., 2021; Prenter et al., 1994), that is, higher fecundity. Males may thus benefit from investing more in the guarding of larger than smaller females (e.g. Knox & Scott, 2006). Last male sperm precedence, when the last mated male fertilises most of a female's eggs (Boggs & Watt, 1981; Parker, 1970; Simmons, 2002; Sims, 1979), but see Tregenza & Wedell, 2002), is common in insects, thus male benefits from postcopulatory mate‐guarding are further enhanced (Alcock, 1994).

Guarded females may benefit from avoiding further harassment or injuries from other males (Dickinson & Rutowski, 1989; Kawagoe et al., 2001; Nilakhe, 1977; Orr, 1999; Thornhill & Alcock, 1983). However, being guarded may deprive females from multiple matings. The consequences of reduced polyandry may be the decreased amount of resources such as spermatophores from other males (Marshall & McNeil, 1989), decreased genetic variability among progeny (Jennions & Petrie, 2000) and less opportunities for postcopulatory mate choice (Firman et al., 2017).

The extension of guarding in time without the males' presence may pay off (King & Fischer, 2005), if fitness is enhanced by guarding and guarding is time‐ and/or resource‐consuming due to lost mating, resting and feeding opportunities. Mating plugs are postcopulatory devices considered to impede or block the females from future mating and are inserted into the female copulatory organ (Stockley et al., 2020). Mating plugs are taxonomically widespread, described from nematodes (Timmermeyer et al., 2010) to primates (Danzy et al., 2009).

In most lepidopteran taxa, internal plugs are common (Matsumoto & Suzuki, 1995; Orr, 1995), while in two butterfly families, Nymphalidae and Papilionidae, large, structured, external, species‐specific mate‐guarding devices, called sphragides (singular: sphragis), evolved independently (Carvalho et al., 2017, 2019). Sphragides are secreted by males, cover the female copulatory opening and may persist on the females throughout their postcopulatory life (Carvalho et al., 2017; Matsumoto & Suzuki, 1995; Orr, 1995). These devices do not block oviposition since most lepidopterans are ditrysian, that is, the copulatory opening is separated from the oopore (Scoble, 1992).

Although the study of sphragides, including arguments on how these prevent butterfly remating, has been started more than a century ago (e.g. Bryk, 1918, 1919; Marshall, 1901), data on within‐species size and morphological variation are scarce (Carvalho et al., 2019; Gór et al., 2023). To our best knowledge, quantitative studies on how this variation impacts the guarding devices' persistence on females are absent to date. Gór et al. (2023) found conspicuously large size and morphological variation of mate‐guarding devices in a single population of Clouded Apollo (*Parnassius mnemosyne*, L.) butterflies. They termed the varieties of the externally visible male‐produced devices that may impede the female's future mating as a Copulatory opening APpendix (CAP). CAPs in this species consist of three types, the filaments, the stopple and the shield. Filaments are small threads found in the female copulatory opening. Stopples are a little larger appendices that, in contrast to filaments, cover the opening entirely. Filaments and stopples are together named as small CAPs. The shield is a warped sheet built on top of a stopple, assumed to prevent stopple removal by rival males and is much larger than small CAPs (Gór et al., 2023). The shield approximately corresponds to the term sphragis (sensu Carvalho et al., 2017; see also Orr, 1995). Such a large morphological and size variation may allow studies to elucidate among‐male and between‐sex dynamics of sexual conflict over mating, as well as the evolution of mating systems with large sphragides.

Our aim was to study CAP‐type replacements within females during their lifetime and to reveal how male investment into different CAPs was (i) related to securing paternity, (ii) associated with female quality and (iii) with actual adult sex ratio. We could not directly measure paternity in live specimens, hence we assumed that paternity was positively related to CAP persistence on the females. Gór et al. (2023) hypothesised that, since easier to remove, small CAPs were less effective in mate‐guarding than the much larger shields (i.e. sphragides). To assess this hypothesis in Clouded Apollo females of a natural population, we estimated CAP replacement risks, using multistate survival models, in an extensive observational study through 6 years based on mark‐recapture. We predicted that small CAPs had higher risks of being lost or replaced by males during a female's life than shields. Furthermore, we investigated how replacement risk was associated with female body size and adult sex ratio. We presumed that larger females were more prone to receive shields than small CAPs since they could be worthier for males due to higher fecundity, and that male‐biased sex ratios could be associated with larger CAPs due to high‐level male competition for mating.

## 2 METHODS

### 2.1 Study species

The Clouded Apollo is a Eurasian butterfly depending on open habitats within deciduous forests (Konvička & Kuras, 1999; Meier et al., 2005; Weiss, 1999). It is univoltine, and flies from late April to the beginning of June in Hungary (Gergely et al., 2018), thus adult generations do not overlap. The egg is in the overwintering stage (Bergström, 2005) and larvae feed in the spring on *Corydalis* DC. (Papaveraceae) species. The sexes are easy to distinguish, since in males, the dorsal side of the thorax and abdomen are densely covered with hair, while these are almost bald in females. Females also have yellow scales on the sides of their abdomen and the back of the head, absent in males. Adults spend much time feeding on nectar plants (Konvička & Kuras, 1999; Szigeti et al., 2018; Vojnits & Ács, 2000). Clouded Apollos are protandrous, that is, males on average emerge earlier than females during the flight period (Szigeti et al., 2019; Vlašánek & Konvička, 2009). Males often patrol seeking females whom they usually force to mate and a CAP may be formed towards the end of mating (Gór et al., 2023). Mated females lay eggs several times during their life (Meglécz et al., 1999, authors' observation).

### 2.2 Study site and period

We carried out fieldwork at Hegyesd, a 0.5 ha colline meadow, surrounded by a Turkey oak *Quercus cerris* L. forest (Figures S11 and S12) in the Visegrádi‐hegység, Hungary, Central Europe (47.756411, 19.047897, at 295 m a.s.l.), between 2015 and 2020. Observations began when the first Clouded Apollo adults appeared and lasted until the last individual was on the wing (Table S1). We sampled butterflies between 9 AM and 6 PM on all days of the Clouded Apollo's flight period, as weather permitted.

### 2.3 Sampling

Mark‐release‐recapture (MRR) was used to survey the population. We aimed to capture all unmarked butterflies with a butterfly net. We marked them individually with a colour combination on both forewing tips with edding® paint markers, gave an identification number on both hindwings and marked new shields with black dots (edding® OH permanent marker; both inside, for better persistence and outside, for better visibility, of the shield wall) and then released them (Szigeti et al., 2018). We monitored the meadow regularly throughout the day, recorded marked females and checked markings on the shields. Furthermore, we attempted to capture all marked females with unmarked shields, as well as all marked females without shields once a day.

For the survey, observers followed the same routes which had been systematically distributed in the meadow to reduce trampling (Szigeti et al., 2016). As it is a small, closed population, we assumed that butterflies were captured soon after their eclosion and their detectability did not vary among individuals and through time.

### 2.4 Copulatory opening APpendix (CAP) types

**FIGURE 1:**
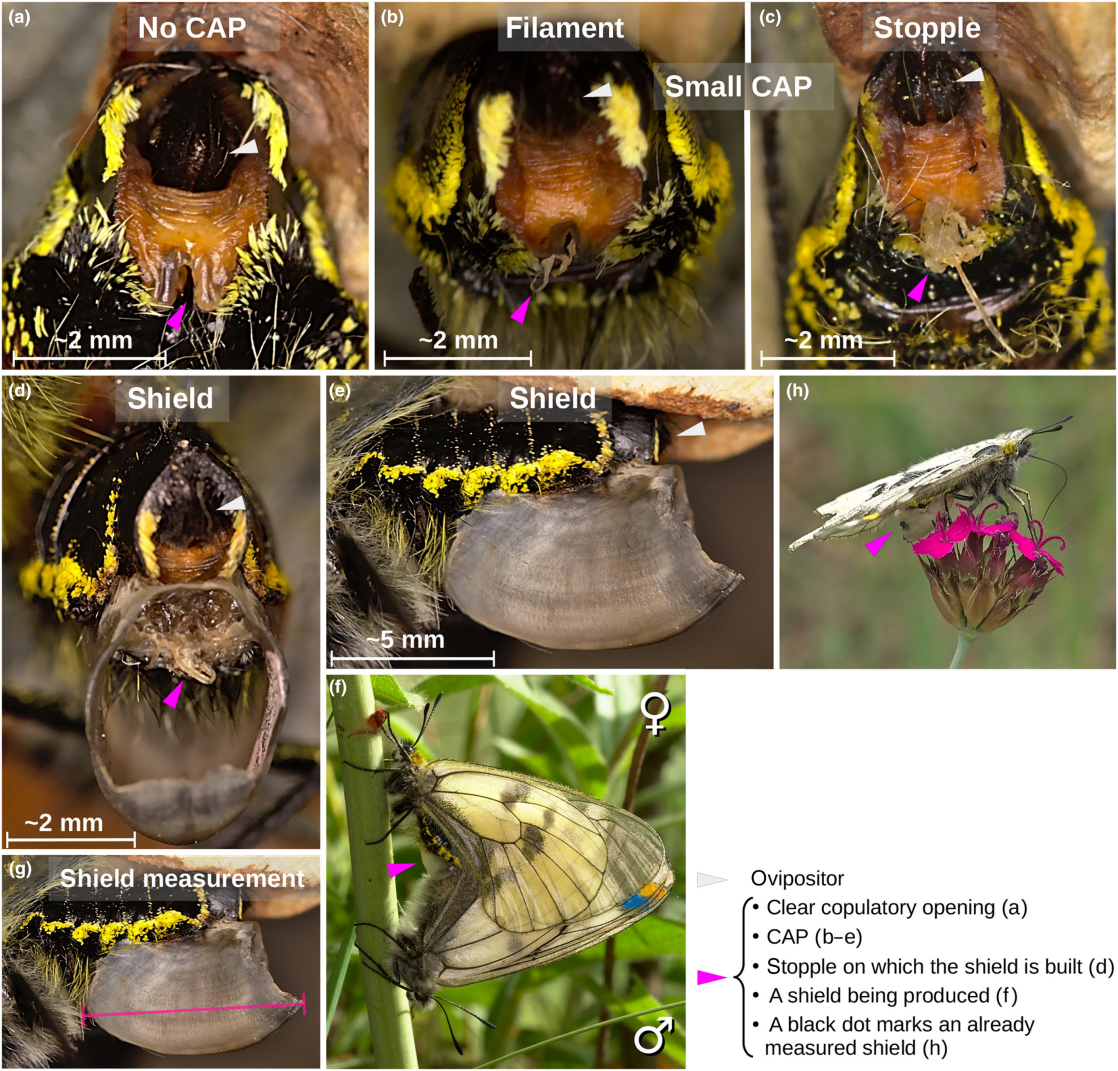
Female Copulatory opening APpendix (CAP) types (a–e) in Clouded Apollo butterflies. A mating pair with a shield being produced (f). The length measurement of the CAP‐type shield (g). A female with a marked shield (h). The copulatory opening is free, no CAP can be observed in virgin or CAP‐lost females (a). Small, thread‐like filaments (b), small, compact stopples (c; b, c together is called a small CAP) or large shields (i.e. sphragides; d, e) may be produced by males during mating (f). All CAPs leave the ovipositor free (b–e). The shield is built over a large stopple, the stopple being fixed in the female copulatory opening (d). Colour dots on the male's forewing are individual markers. Note sexual dimorphism in body colouration and hairiness (f). Shield length was measured as the largest anterior–posterior distance with callipers (g). A feeding female's black dot on the shield's posterior shows that this shield had already been measured; the dot can be seen with binoculars from a distance (h). Views: posterior‐ventral (a–c), posterior (d) and left lateral (e). Light grey arrowheads show the ovipositor (a–e), the magenta arrowheads indicate the clear copulatory opening (a) or the respective CAP (b, c, f) or the stopple on which the shield is built (d), or the black dot marker on the shield's posterior tip (h). All pictures were taken by JK; butterflies captured for a–e, g were later released.

Virgin females start their life with no CAP, that is, no appendix can be observed externally in the copulatory opening (Figure 1a). Although no appendix is present, for convenience, we define this category as a CAP‐type in this study. Some females receive a small CAP when mating (Figure 1b,c). The small CAP consists of two morphologically distinct types, the filament, a thin, thread‐like device not covering the copulatory opening (Figure 1b) and the stopple, a small, compact device covering the opening externally (Figure 1c). Both filaments and stopples vary in size and shape (Gór et al., 2023). Other females may receive a shield (i.e. sphragis, Carvalho et al., 2017; Figure 1d–f). The shield is a sheet warped around and attached to a stopple (Figure 1d). Thus, the stopple is believed to be produced first, then the shield built around it if the male is able and willing to invest more (Gór et al., 2023). Small CAPs are much smaller than shields. Both types can be lost without replacement, so no CAP does not inform on virginity. CAP‐types (small CAP (filament or stopple) vs. shield) could be either spontaneously lost or males may replace them with either the same or different CAP‐types during a female's life (Gór et al., 2023). CAPs do not block egg laying in Clouded Apollos (ditrysia; fig. 2F in Gór et al., 2023; Figure 1b–e).

### 2.5 Measurements

We measured shield length, twice consecutively, with callipers with thinned jaws for better access, and used the means of the two measurements in the analyses. Length was the longest distance between the anterior tip and the ventral posterior end of the shield (Figure 1g). Shield length ranged from 3.775 to 13.000 mm (Gór et al., 2023), and we used it as a proxy for male material investment into a shield. We also took photo macrographs from different angles on the area around the copulatory opening of non‐shielded females. This informed us on whether a female was bearing a CAP or not. Furthermore, it provided a minimum estimate on within‐individual small CAP replacements based on unique CAP morphology (Gór et al., 2023). Although we cannot claim that consecutive small CAPs with a similar shape on the same female were really the same CAPs, differences in shape signal subsequent mating events.

Since matings were scarcely observed, we could associate body size with the CAPs produced only for females. Thorax width was measured at first capture, then we attempted to recapture all marked individuals to repeat thorax width measurements every third day. It was measured twice at each occasion with callipers to 0.1 mm. Thorax size is related to the actual amount of muscles important for butterfly flight (Stjernholm et al., 2005). In Clouded Apollos, thorax width shrinks over an individual's life with declining body mass (i.e. phenotypic senescence, Pásztor et al., 2022), thus both are related to the actual quality of an individual.

We also recorded three scaled photographs from the anterior view of the head at first capture, then from these photos we measured head width, the largest distance between the lateral edges of the eyes, the widest part of the butterfly head, twice from each photograph. Head width is considered a proxy of body size in insects (Dahlsjö, 2016; Hagen & Dupont, 2013), including Lepidoptera (Mo et al., 2013), associated with fecundity (Schmidt & Blume, 1973). Since it does not change over adult life, it reflects an age‐independent quality of an individual.

Photo macrographs on mate‐guarding devices, that is, CAPs, female genitals as well as heads were captured on live individuals fixed on a small board for easy handling. Butterflies were laid on their backs, with wings in a relaxed position and covered with plastic plates, then clipped to the board. The board had a small depression for the dorsal thorax to fit in, in order to prevent tension in wing muscles. Photographs were taken with a Nikon D7000 camera mounted on a tripod, with either a 60 mm f/2.8G ED AF‐S Micro NIKKOR or a Sigma 105 mm f/2.8 EX DG OS MACRO lens. Measurements on photographs were done with Fiji ‐ ImageJ (Schneider et al., 2012). Later in the analysis, the mean of all six measurements was used for each individual.

Other dimensions, such as shield height and width (Gór et al., 2023), body mass, wing length (Pásztor et al., 2022) and proboscis length (Szigeti et al., 2020) were also measured (see detailed methodology therein), however, these were omitted from the results due to their non‐significant effect. All measurements in the field were carried out by JK.

### 2.6 Variables

For the analyses, we distinguished female states as no CAP, small CAP, shield and disappeared (i.e. the females from the population). We use the term female state only for modelling and discussing model results. Note that this term includes CAP‐types, the term we use to describe the mate‐guarding devices (or their absence), but is not equivalent to that. We use the term transition for changes among female states, including if a state is followed by the same state within a female (e.g. a small CAP followed by another small CAP). Matings between two consecutive no CAP observations were not detected, that is, no transitions could be found between two no CAPs. The disappearance of a female is referred to as disappeared because it might mean death, emigration or that we simply failed to further observe her. A previous mark‐release‐recapture study on this population between 2016 and 2019 using Jolly‐Seber models estimated that ~90% of the individuals in this relatively closed population had been captured at least once in each year (Zorkóczy, 2020), suggesting that individuals present in the population were mostly detected.

**FIGURE 2:**
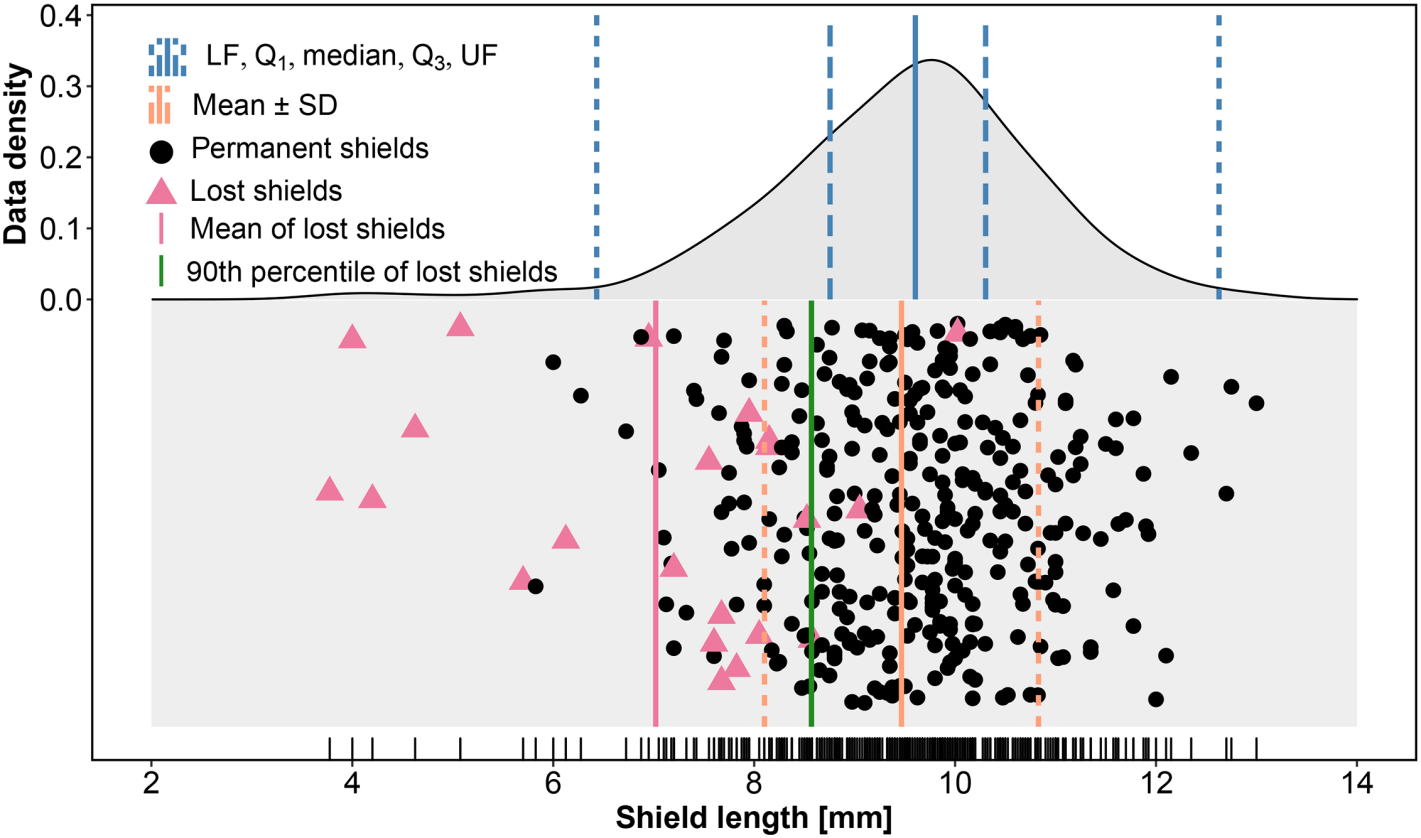
The distribution of shield lengths for the 6 years combined in Clouded Apollo females. Note the difference between the lost (red triangle) and the permanent (black dot) shields. Blue vertical lines show the lower fence (dashed), first quartile (long dashed), median (solid), third quartile (long dashed) and the upper fence (dashed) for all shields. Orange lines represent the mean (solid) and standard deviation (dashed) of permanent shields. The vertical red line shows the mean of lost shields and the vertical green line indicates the 90th percentile of lost shields. Above this value, shields are long, below, shields are short. Data points are jittered along the y‐axis for better visibility. We observed a total of 23 shield losses, but only 22 shields were measured.

We transformed shield length to a binary variable; below the 90th percentile of lost shields (8.565 mm; 68th percentile among non‐lost shields), we defined the shield as short, above it, as long (Figure 2).

We used the exact or the closest (±3 days) thorax width values to the day of the transition. To provide an estimate of the actual condition of a female unrelated to its natal body size (i.e. size at emergence), we used the natural logarithm of the thorax width to head width ratio, that is, ln(thorax width) – ln(head width), calculated for every time point for the transitions.

We calculated shield‐to‐CAP ratios for each day, the number of shields observed per day divided by the total number of daily CAPs. We also calculated daily adult sex ratios (ASR, Kvarnemo & Ahnesjö, 2002) and used them as either a continuous or a binary variable. In the latter, the population was considered female‐biased below, and male‐biased above 0.5.

### 2.7 Statistical analyses

We evaluated all data in the R statistical environment (version 4.2.3, R Core Team, 2023). We plotted annual CAP‐type event histories (Carey et al., 1998, 2006) to present seasonal CAP variation across females and years. For plotting shield lengths, CAP‐type event histories, female state proportions and ASRs/shield‐to‐CAP ratios, we used the package ‘ggplot2’ (version 3.4.2, Wickham, 2016).

A semiparametric multistate survival model (Putter et al., 2007) was applied to assess transition risks between the consecutive observations of individual females. Briefly, multistate survival models quantify time‐dependent transition probabilities between well‐defined states of individuals. The hazard in a multistate survival model is approximately the probability of transition from the current state to a subsequent state during unit time, 1 day in our model. The hazard depends on the actual follow‐up time when the transition happens. There are unique hazard submodels for each possible transition between states. In a semiparametric multistate model, the hazards are given as a product of a nonparametric time‐dependent function of a general form and a fully parametric multiplicative expression. This expression depends on certain risk factors or covariates and the corresponding relative risks being the parameters of the model.

Similar annual patterns in CAP frequencies (see Section 3.1) allowed the analysis with the years pooled. In the model, females could have four states at any given observation, no CAP, small CAP, shield and disappeared. Where no CAP was not the first observed female state (344 females out of 492), we added 1 day of no CAP to female life preceding the first detection, since females start their life virgin, as no CAP. However, most females are first detected with CAPs, that is, when already mated, hence we assumed that they had mated very soon after emergence. We pooled the CAP‐types filament and stopple into small CAP since these are much smaller than the shortest shields (Figure 1b–e), therefore, we assume that, compared to shields, male investment and their capacity to prevent future matings are similarly small. Small CAPs and shields were sometimes replaced with the same CAP‐type in an individual between two consecutive observations. However, multistate models cannot recognise this pattern of change as a state transition. To overcome this problem, we introduced dummy states, for example, a shield could transition to the dummy then the dummy into another shield, with the time ‘spent’ in the dummy state closing to zero. This resulted in slightly different frequencies of model transitions compared to the number of replacements that we observed in the field.

We intended to evaluate the differences in risks of small CAP and shield losses. However, we found interactions between relative risks and time in our multistate model. Therefore, to simplify the evaluation, we computed 99.16% confidence intervals around the cumulative hazards of the small CAP and shield losses. This allows for multiple comparisons of the small CAP and shield loss risks at a 5% adjusted significance level, using Bonferroni's method. For better visualisation, we transformed transition hazards from the model as ln(hazard × 10 + 1) for the cumulative hazards plot.

Within the framework of this complex model, we fitted nonparametric cause‐specific hazard functions to each transition between states to estimate instantaneous transition risks at each time point measured since the onset of the follow‐up. The package ‘mstate’ (version 0.3.2, de Wreede et al., 2011) was applied to fit the multistate survival model. We fitted proportional hazard submodels to investigate the differences in relative risks among transitions starting from the same states (e.g. shield to shield and shield to small CAP). To account for time‐dependent changes in transition risks, we included interaction terms between time and transitions. In further submodels, we included (i) the binary shield length as a factor, or (ii) thorax width to head width ratio (female's actual condition controlled for natal size), as well as head width (natal size) as covariates, or (iii) the binary adult sex ratio as a factor. In the submodels containing thorax width to head width ratio or shield length, data were clustered by the individual identification number to obtain proper marginal estimates (Therneau & Grambsch, 2000), because females were measured multiple times and could bear multiple shields during their lifetime. Relative risks (RR) of covariate effects modifying baseline transition hazards were estimated. The nonlinear effect of thorax width to head width ratio was accounted for by including the squares and cubes of this variable in the submodel. Non‐significant terms were then eliminated. The hazard submodels were fitted using a stratified Cox proportional hazards model (Therneau & Grambsch, 2000) with time‐dependent covariates applying the R function coxph from the package ‘survival’ (version 3.5–5, Therneau, 2023). We used the Breslow method when compiling the submodels. To test the proportional hazards assumption, we used Schoenfeld residuals (Therneau & Grambsch, 2000). Martingale residuals and deviance residuals (Therneau & Grambsch, 2000) were also inspected to detect potential influential points causing bias.

To investigate the time‐dependent relationship between daily shield‐to‐CAP ratio and daily adult sex ratio (ASR) as a continuous variable, we performed a time series analysis. We excluded (i) days where ratios were equal either to zero or one (when only shields or small CAPs, or males or females were present in the population on a specific day) and (ii) influential points (days 25 May 2015 and 29 May 2015), as well as (iii) the entire year 2020 due to the very low number of small CAPs (Figures S7 and S8). Then we pooled years (2015–2019) and used the cross‐correlation function (CCF; package ‘astsa’, version 2.0, Stoffer & Poison, 2023) to compute the correlation between the time series of the two ratios. We transformed these using a natural logarithm, then calculated the difference between subsequent daily values as ln(ratio[*t*]) – ln(ratio[*t*−1]). This was necessary to be able to investigate the percentage change in ratios between days. In the CCF, the three‐day lag provided the largest absolute value of the correlation coefficient (Figure S9). We fitted a generalised linear mixed effects model (GLMM; package ‘nlme’, version 3.1‐162, Pinheiro & Bates, 2023) for the logarithm of daily shield‐to‐CAP ratio using the 3‐day time lag of the logarithm of adult sex ratio as the explanatory variable, assuming first‐order autoregressive (AR1) residuals. The year of observation was included as a random intercept. Based on this model, we estimated elasticity (Sydsæter et al., 2016) between shield‐to‐CAP ratio and the adult sex ratio 3 days earlier.

## 3 RESULTS

Between 2015 and 2020 we observed a total number of 492 Clouded Apollo females. The duration of the flight periods and the number of females varied considerably across the years (Table S1). The longest season was in 2019 with 45 days, the shortest was in 2018 with 26 days. The most females were present in 2018 with 116 individuals, the lowest number of females was in 2020 with 34 individuals when the total number of Clouded Apollos dropped drastically.

### 3.1 CAP variation

We observed a total of 154 cases of no CAPs, 127 filaments, 120 stopples (247 small CAPs) and 356 shields (23 lost) between 2015 and 2020 (Table 1). The number of these CAP types varied across years, as well as the annual proportion of shielded among all females, with a minimum of 67.9% and a maximum of 80.0% (Table 1).

**TABLE 1:** Frequencies of Clouded Apollo Copulatory opening APpendix (CAP) types over 6 years.

Year	*N*_no CAPs_	*N*_filaments_	*N*_stopples_	*N*_small CAPs_	*N*_shields_	%_shielded females_	*N*_lost shields_
2015	18 (18)	20 (20)	21 (17)	41 (29)	61 (57)	67.9	7
2016	31 (31)	31 (22)	32 (23)	63 (35)	62 (62)	70.5	5
2017	26 (26)	21 (14)	16 (15)	37 (22)	63 (62)	71.3	2
2018	30 (30)	30 (19)	20 (17)	50 (30)	87 (85)	80.0	7
2019	35 (35)	23 (15)	27 (17)	50 (25)	58 (57)	68.7	2
2020	14 (14)	2 (1)	4 (4)	6 (4)	25 (25)	74.0	0
2015–2020	154 (154)	127 (91)	120 (93)	247 (145)	356 (348)	73.0	23

*Note*: Small CAPs consist of filaments and stopples. The number of females observed with a specific CAP‐type is shown in parentheses. We also provide the percentage of shielded out of all females and the number of shields lost. The no CAPs column shows cases when no CAP females were actually observed. A female could be observed with several different or similar consecutive CAPs during her life.

**FIGURE 3:**
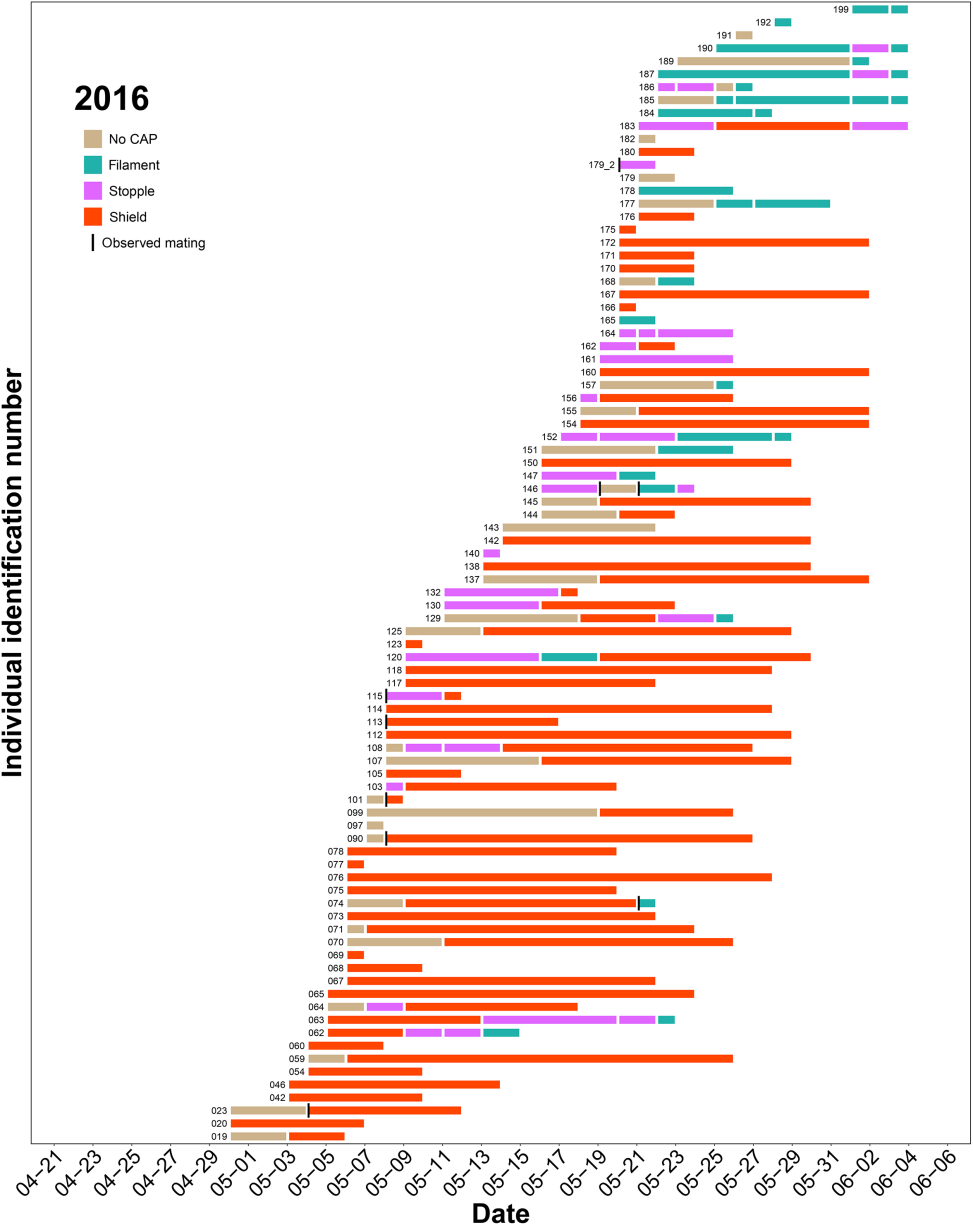
Copulatory opening APpendix (CAP) event histories in 2016; actual observations on Clouded Apollo females. Each horizontal row (ordered by the annual individual identification numbers from bottom to top) represents the history of a female butterfly and row length shows the individuals' observation period. Colour‐coded segments indicate the different CAP types. Vertical black bars show the observed matings. See Figures S1–S6 for each year.

Although we found considerable annual variation in CAP‐type event histories, probably due to the variable phenologies, a general, annual pattern emerges (Figure 3, Figures S1–S6). Females start their life with no CAP, however, we usually find them already mated. At the beginning of the flight period, shielded females are more abundant than later, when the occurrence of small CAPs becomes predominant (Figure 3, Figures S1–S6). Furthermore, these figures imply that small CAPs were lost much more frequently and lasted for a shorter period than shields (for further evaluation see the CAP‐persistence section).

**FIGURE 4:**
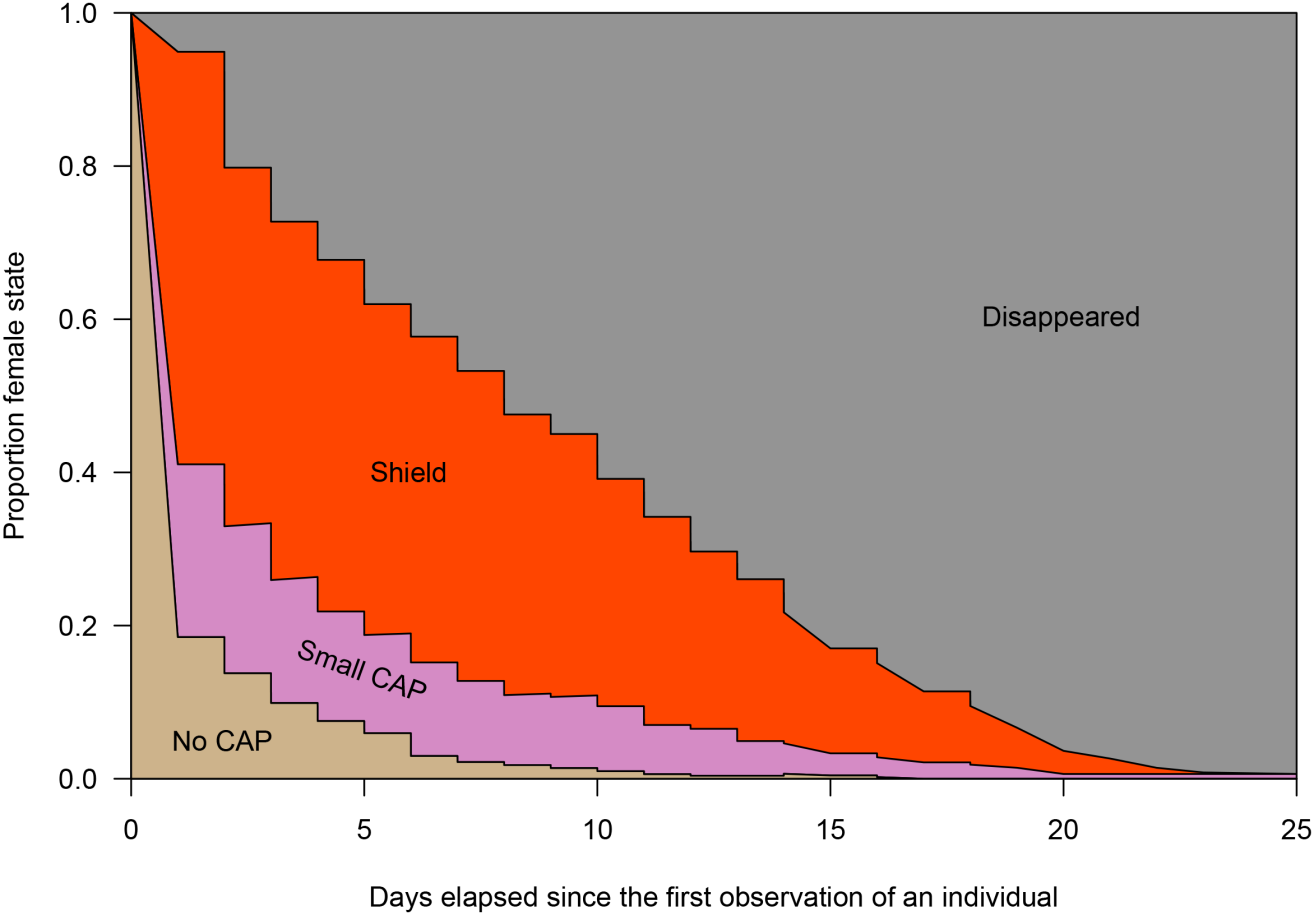
The proportion of each female state over the Clouded Apollo females' life, with the years pooled, based on the fitted semiparametric multistate survival model. Although most females were first caught already bearing a Copulatory opening APpendix (CAP), we assumed that all started their lives in the no CAP state.

Based on the fitted semiparametric multistate survival model, we plotted the proportions of female states according to the time elapsed since the first observations of the individuals (Figure 4). Females start their life as no CAP, the proportion of which decreased very steeply in the first few days. During the life of females, there was always a small proportion of those in the state of no CAP or small CAP, and a larger proportion bearing shields. The proportion of individuals bearing a shield decreased more strongly in the last days of the individuals' life, while the proportion of females with small CAP decreased less steeply (Figure 4).

### 3.2 CAP‐transitions

**FIGURE 5:**
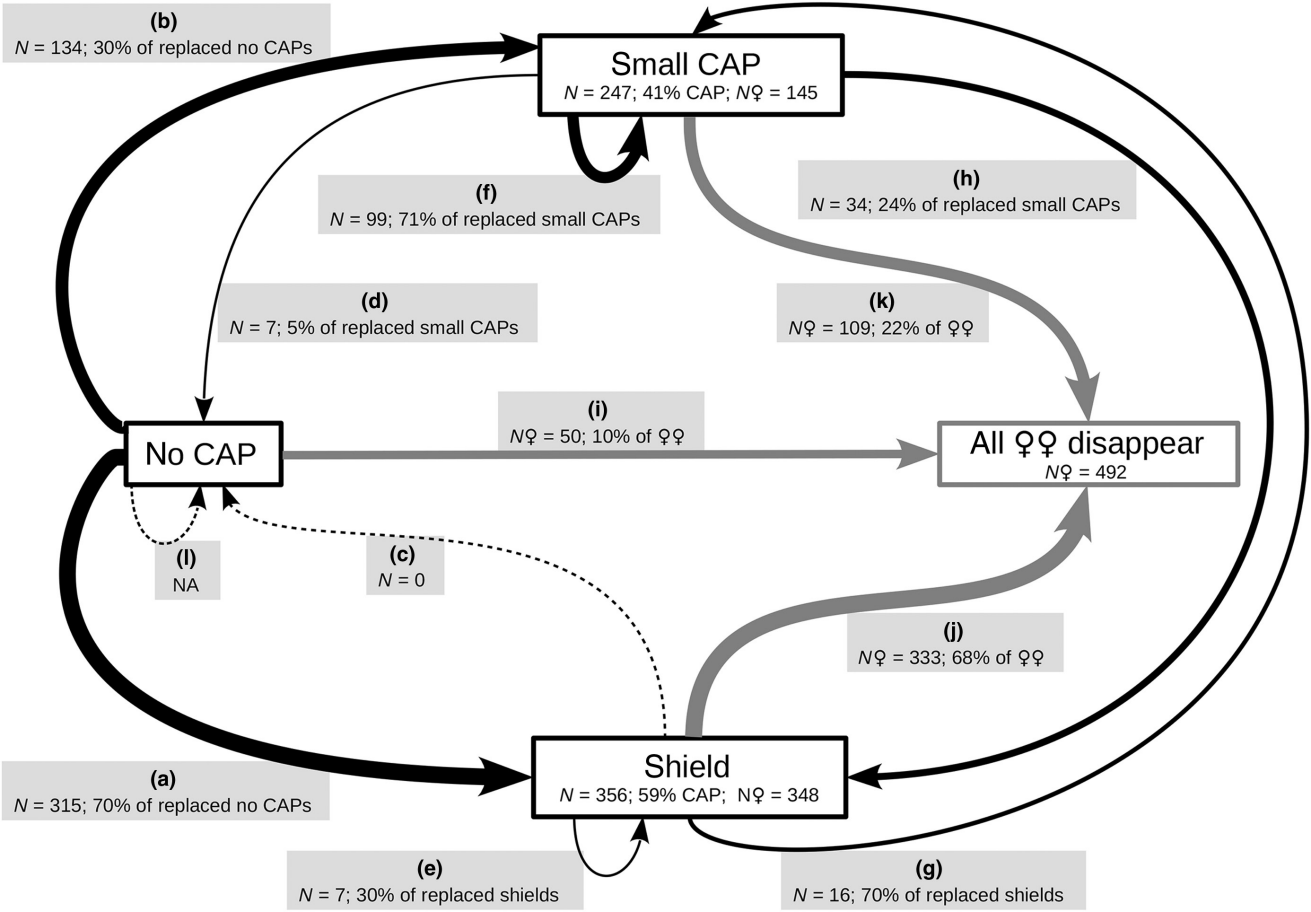
Observed female state transitions (arrows); transitions among Copulatory opening APpendices (CAP) (black arrows, a–h) and female disappearance from the population (grey arrows, i–k) in Clouded Apollo butterflies, 2015–2020. Framed boxes denote female states. The number of states of the entire sample, the percentage of the state relative to all CAPs and the number of females observed with the respective states are shown. We did not provide statistics for no CAPs, since all females were assumed to start their life in this state. The box with all females disappeared (grey frame) shows our entire sample. The boxes attached to black arrows (a–h) show the observed transitions between female states with respective sample sizes and the percentage of transitions within the state the arrow originates from. We did not observe shield loss without replacement (c, dashed line), only prior to this study. Transitions from no CAP to no CAP could not be investigated (l; not available: NA). The boxes attached to grey arrows (i–k) show the number of females and the percentage of their last observed state among all females. The line width of arrows is proportional to the total number of transitions observed, except c & l (width = ln(% total transitions + 1); the exact formula was selected upon best visual presentation).

We observed nearly all possible transitions between states, except the transition from shield to no CAP (Figure 5), a transition observed once in this population out of this study's period in 2014.

The number of each transition varied across years (Table S2). In every year, the most frequent transitions were females with no CAP receiving a shield (315 cases), and shielded females disappearing from the population (333 cases). Scarce transitions were when a small CAP was followed by a no CAP state (7 cases), and when a shield was replaced with a small CAP (16 cases) or another shield (7 cases) (Table S2). There were cases when transitions of the same type happened several times in the same female, that is, (i) a no CAP state followed by a small CAP occurred twice in four females and (ii) a small CAP replaced with another small CAP occurred 2–4 times in 10 females (Table S2).

### 3.3 CAP‐persistence

**FIGURE 6:**
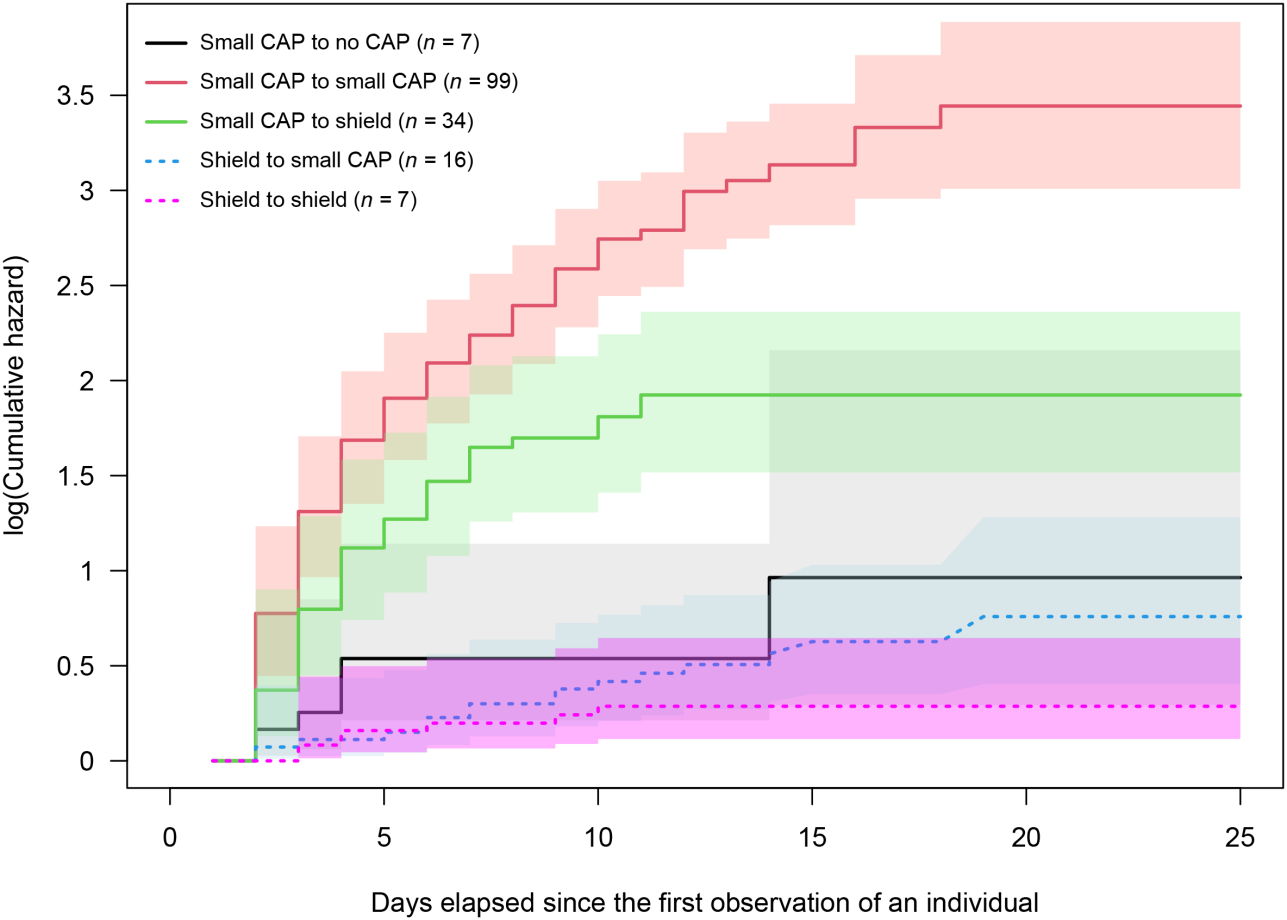
Cumulative hazards of small CAP (Copulatory opening APpendix) and shield losses in Clouded Apollo females from the semiparametric multistate survival model at a given observation time. Lines show the hazard functions, shadings are the 99.16% confidence intervals of the functions.

The lack of overlap between confidence regions indicates that the average risk over time to lose a small CAP (solid red and green lines) was significantly higher than the average risk over time of losing a shield (dotted blue and magenta lines) during the entire range of the individuals' observation period (Figure 6). The transition from small CAP to no CAP (solid black line) is a poor estimate (see wide grey confidence region) and did not significantly differ from losing a shield (Figure 6).

### 3.4 Proportional hazard submodels

In transitions starting from the no CAP state (*n*(females) = 492; *n*(transition) = 499), the risk of transitioning to a shield (relative risk; RR = 2.38; *p* < .001; CI: 1.95, 2.92) was 2.38× larger, and the risk of disappearance at a given time point (RR = 0.36; *p* < .001; CI: 0.26, 0.51) was 0.36× smaller than transitioning to a small CAP. Time interactions had no significant effects (*p* = .988 and *p* = .127).

In transitions starting from a small CAP (*n*(females) = 145; *n*(transition) = 247), the risk of transitioning to another small CAP (RR = 2.98; *p* < .001; CI: 1.86, 4.77) was at least 2.98× larger, and the risk of disappearing at a given time point (RR = 1.77; *p* = .018; CI: 1.11, 2.85) was at least 1.77× larger than transitioning to a shield. The time interaction was significant for the transition from small CAP to small CAP (RR = 1.08; *p* = .049; CI: 1.00, 1.17) and for the transition from small CAP to disappearance (RR = 1.16; *p* < .001; CI: 1.08, 1.25) which resulted an increase in the relative risks over time.

In transitions starting from a shield (*n*(females) = 348; *n*(transition) = 356), the risk of disappearance at a given time point (RR = 21.43; *p* < .001; CI: 12.84, 35.76) was 21.43× larger than transitioning to a small CAP, meaning that shields usually persist until the end of an individual's life. There was no significant difference in the risk between transitioning from a shield to a small CAP and another shield (RR = 0.82; *p* = .763; CI: 0.22, 3.01). Time interactions were not significant (*p* = .581 and *p* = .447).

### 3.5 Models with covariates

According to the submodel on the relationship between shield loss and shield size (*n*(females) = 348; *n*(transition) = 1205), the risk of transitioning from a shield to a small CAP for short shields was 27.56× larger than for the long shields (RR = 27.56; *p* < .001; CI: 5.83, 130.40). In addition, transitioning from a shield to another shield for short shields was 25.42× larger than for long shields (RR = 25.42; *p* = .003; CI: 2.96, 218.60). Shield height and width were not significantly related to shield loss (results not presented). In summary, shorter shields were more prone to be lost than longer ones.

According to the submodel on the relationship between transitions and female sizes (*n*(females) = 492; *n*(transition) = 1190), the risk of receiving a shield either after a no CAP state (RR = 3.70; *p* = .012; CI: 1.34, 10.23) or a small CAP (RR = 503.70; *p* = .002; CI: 9.06, 2.80 × 10^4^) was significantly higher in females with wider thoraces than in females with narrower thoraces; that is, females with wider thoraces are more prone to receive shields than narrower females. Moreover, the risk of disappearance at a given time point either after bearing a small CAP (RR = 7.16 × 10^−4^; *p* < .001; CI: 7.56 × 10^−5^, 6.79 × 10^−3^) or a shield (RR = 0.05; *p* < .001; CI: 0.01, 0.18) was significantly lower in females with wider thoraces than in females with narrower thoraces.

Similarly, the risk of receiving a shield after a no CAP state was significantly higher in females with wider heads than in females with narrower heads (RR = 3.99; *p* < .001; CI: 1.89, 8.42). However, no significant relationship was found between the risk of receiving a shield after a small CAP and female head width (RR = 16.75; *p* = .133; CI: 0.42, 662.50).

Moreover, the risk of disappearance at a given time point either after a no CAP state (RR = 9.68 × 10^−3^; *p* < .001; CI: 6.56 × 10^−4^, 1.43 × 10^−1^) or after bearing a small CAP (RR = 0.15; *p* < .037; CI: 0.02, 0.89) or a shield (RR = 0.35; *p* < .026; CI: 0.14, 0.88) was significantly lower in females with wider heads than in females with narrower heads.

Female body mass, wing and proboscis length were not significantly related to transitions in the female state (results not presented). Taken together, both females with wider thoraces, when controlled for head width, and females with wider heads were more prone to receive shields and less prone to disappear at a given time point than females with narrow thoraces, while other measures of body size had no significant effects.

The submodel on the relationship between transitions and ASR (*n*(females) = 492; *n*(transition) = 1206) showed that the risk of receiving a shield either after a no CAP state (RR = 3.67; *p* < .001; CI: 2.21, 6.09) or a small CAP (RR = 7.04; *p* = .001; CI: 2.12, 23.41) was higher when the population was male‐biased.

Furthermore, the risk of disappearance at a given time point either after a no CAP state (RR = 0.38; *p* = .003; CI: 0.21, 0.72) or after bearing a small CAP (RR = 0.31; *p* < .001; CI: 0.20, 0.47) or a shield (RR = 0.41; *p* < .001; CI: 0.29, 0.58) was lower when the population was male‐biased. In addition, the risk of receiving a small CAP after a no CAP state (RR = 0.49; *p* < .001; CI: 0.33, 0.72) was also lower when the population was male‐biased.

### 3.6 Time series analysis

**FIGURE 7:**
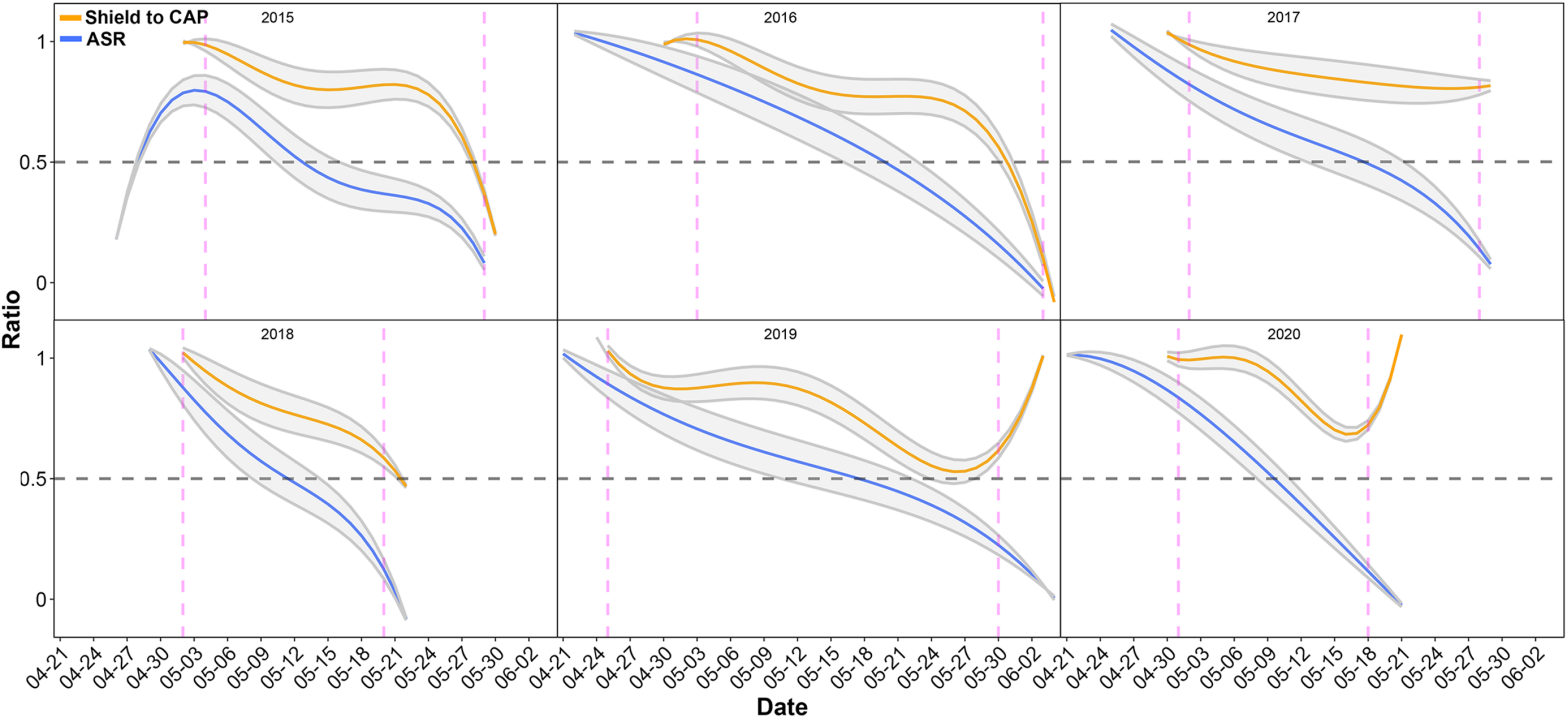
Shield‐to‐CAP (Copulatory opening APpendix) ratio (orange curves) and adult sex ratio (ASR, blue curves) changes over the flight periods in 6 years in Clouded Apollos. Grey shading width is proportional to the number of individuals (width = ln(daily *n*_ind._/50); exact formula was selected upon best visual presentation). The areas between the vertical dashed lines are intervals with at least 5 females observed in the population each day. We think that out of these intervals, ratio estimates could be severely biased. The horizontal dashed lines show equal numbers of shields and small CAPs or males and females for a given day; at 0 only small CAPs or females, at 1 only shields or males were present in the population.

Both the proportion of shields and the proportion of males in the population decreased with time (Figure 7).

In line with Figure 7, the change in shield‐to‐CAP ratio was significantly related to the change in ASR with a three‐day lag (*n*(observations) = 118; elasticity = 0.19; *p* = .003; CI: 0.07, 0.32; GLMM). Here, elasticity is interpreted as a 1% decrease in daily ASR involves, on average, a 0.19% decrease in the daily shield‐to‐CAP ratio. Year as a random factor explained only an extremely small proportion of the total variance (0.89%).

## 4 DISCUSSION

By examining 492 Clouded Apollo females we found that (i) shields were less prone to be lost than small CAPs and (ii) longer shields were less prone to be lost than shorter ones. (iii) Females with wider thoraces were more prone to receive shields than narrower females and (iv) females with narrower thoraces had a higher risk to disappear at a given time point than wider ones. (v) Females were more prone to receive a shield when the population was male‐biased and receive a small CAP or disappear when the population was female‐biased; (vi) ASR and the ratio of shields decreased with time during the flight period and the decrease in ASR was followed by the decrease of the shield‐to‐CAP ratio 3 days later.

### 4.1 CAP‐type and size

We found that shields were more common and much less prone to be lost than small CAPs and usually persisted during the whole life of females. This indicates that shields were more efficient devices in securing paternity than small CAPs. Furthermore, longer shields were more prone to persist than shorter ones. These suggest that (i) the more a male invests in CAPs, the more prone he is to secure paternity and (ii) the final outcome of CAP production (i.e. CAP‐type and size) takes place on a more or less continuous scale of investment (Gór et al., 2023). The resources males can actually allocate in mate‐guarding may heavily impact success, and this would, in turn, depend on actual male quality, such as body size (Schöfl & Taborsky, 2002), age (Pásztor et al., 2022), as well as body reserves (Stjernholm & Karlsson, 2000). Male investment is supposed to be constrained through production capacity. ‘Incomplete sphragides’, structures attached to the female copulatory opening, but reduced in size compared to the species‐specific sphragis (Carvalho et al., 2019), likely equivalent or similar to what we denote as small CAPs (Gór et al., 2023), were produced by males after consecutive matings and attributed to male resource depletion in several butterfly species (Carvalho et al., 2017, 2019; Matsumoto, 1987; Orr, 1988). Furthermore, a comparison of six papilionid butterflies found that species producing larger sphragides produce smaller spermatophores suggesting high costs of investing in sphragides (Matsumoto & Suzuki, 1995).

### 4.2 Female quality

Male decision on investment should theoretically depend on female quality, the expected residual reproductive value of the female at the time of mating and the male's expected share of her prospective progeny (Bonduriansky, 2001; Fischer et al., 2008). Males may be able to assess if females are virgins or already mated, and invest accordingly. Its mechanism might be straightforward in the case of female Clouded Apollos with CAPs, that is, anything blocking the vagina means the female has already mated. We have no information if males were able to assess if no CAP females were virgins, and our methods are inappropriate to ascertain female virginity.

Females with both larger actual size (i.e. wider thoraces relative to head width), and larger natal size (i.e. wider heads) were more prone to receive shields than smaller females, while body mass, wing and proboscis length were not related to female states. Thorax size was related to the actual amount of flight muscle mass (Stjernholm et al., 2005). Investment in mate‐guarding increased with increasing female size (Dick & Elwood, 1990; Jormalainen, 1998; Takeshita & Henmi, 2010) and large females were chosen over small ones (Knox & Scott, 2006; Manning, 1975; Shuster, 1981; Thompson & Manning, 1981, but see Jarrige et al., 2016; Mahoney et al., 2017; Schöfl & Taborsky, 2002). Since larger female insects were supposed to be better quality (Gilbert, 1984; Honěk, 1993; Oberhauser, 1997; Okada et al., 2021; Prenter et al., 1994), that is, have higher fecundity, males should benefit from investing more in guarding larger females (e.g. Knox & Scott, 2006). Thus, larger females may receive shields due to their higher residual reproductive value and because shields are more efficient in securing paternity. These imply male preference for large females, size‐dependent investment into guarding and an unknown mechanism of size assessment.

### 4.3 Shield‐to‐CAP ratio and adult sex ratio

Shield‐to‐CAP ratio and adult sex ratio both decreased over the flight period (Figure 7). Similarly, studies investigating the presence or lack of sphragis (shield) found females lacking a sphragis more frequently at the end of the flight period than earlier (*Parnassius mnemosyne*, Vlašánek et al., 2009; Vlašánek & Konvička, 2009; *Parnassius smintheus*, Matter et al., 2004; Roland et al., 2000, but see Auckland et al., 2004 for *Parnassius clodius*). Calabrese et al. (2008) explained the lack of sphragis at the end of the reproductive period with the lack of capable males, termed as matelessness, as ASRs become more and more female‐biased. Our results do not refute the female matelessness hypothesis, rather they emphasise that the lack of a conspicuous large sphragis (shield) does not inform on matelessness. As in many lepidopterans (Teder et al., 2021), all studied *Parnassius* populations were protandrous (Calabrese et al., 2008; Szigeti et al., 2019; Vlašánek et al., 2009; Vlašánek & Konvička, 2009). These imply strongly male‐biased populations at the beginning, less male‐biased or even female‐biased at the end of the flight period, as were also found in another Clouded Apollo population (Vlašánek et al., 2009; Vlašánek & Konvička, 2009. This, together with both sexes living in the flight period later being smaller than those living earlier (Pásztor et al., 2022), thus probably having less reserves, imply that female matelessness might occur in this population at the very end of the flight period.

The seasonal decline of shield‐to‐CAP ratios followed the decline of adult sex ratios with a three‐day lag (Figure 7, Figure S10). The main source of this bias in actual ASR is likely protandry, but further bias towards males may also be because there are more males than females in the Clouded Apollo (authors' unpublished data for a different population, Vlašánek et al., 2009; Vlašánek & Konvička, 2009), as well as in other *Parnassius* populations studied so far (Vlašánek et al., 2009; see also references therein). The intensity of competition for mating among males is expected to be higher with a stronger male bias within the population (Kvarnemo & Ahnesjö, 2002; Weir et al., 2011). Male competition for mating then increases with the male ratio, which may result in increased benefits from mate guarding (Alcock, 1994). In crustaceans, investment in guarding increased with male ratio (Dick & Elwood, 1990; Jormalainen, 1998; Takeshita & Henmi, 2010). Last male sperm precedence (Alcock, 1994; Boggs & Watt, 1981; Clarke & Sheppard, 1962; Labine, 1966; Parker, 1970; Simmons, 2002; Sims, 1979, but see Tregenza & Wedell, 2002), not investigated in Clouded Apollos but common in insects may further explain why males are less prone to invest in shields later in the flight period, when competition for mating as well as the risk of small CAP removal are relatively low.

We investigated adult sex ratios (ASR). In contrast, Alcock (1994) and Kvarnemo and Ahnesjö (2002) suggested that operational sex ratios (OSR), that is, the ratios of males to females willing and able to mate at an actual time point will determine the measure of competition, and ultimately, the benefit from mate‐guarding. In the case of Clouded Apollos, CAP‐type and size influence the female ability to mate, that is, her ‘time‐in’ period (Kvarnemo & Ahnesjö, 2002), therefore, OSR. We do not have any measurable cues to estimate ‘time‐in’ or ‘time‐out’ for males, thus the gap between ASR and OSR estimates cannot be assessed. We predict that ASR underestimates OSR early in the flight period when young, unmated males are abundant and most mated females are shielded. We are unable to predict the ASR‐OSR relationship for the late flight period, with both sexes ageing and females bearing small CAPs being more frequent.

How males could assess the intensity of competition is unknown. One potential explanation would be that patrolling males' encounter rates with competitors and entirely (no CAP) as well as partially (small CAP) available females may influence male decision. Our results suggest that males may not be able to immediately assess the level of competition for mating. Matings usually last several hours and may easily take half a day. This explains a part of the three‐day time lag between shield‐to‐CAP and adult sex ratios, but we are still far from understanding it entirely.

### 4.4 Dynamic changes

The major limitation of this observational study is that we are not able to discern the impacts of different variables changing over the flight period. In the same population as the present study, Clouded Apollo thorax width declined with age in both sexes, and individuals emerging late in the flight period tended to be smaller (Pásztor et al., 2022). Both ageing and small body size are related to less reserves and probably incur low mobility. These may reduce foraging in both sexes, egg‐laying activity in females and patrolling and investing in CAPs in males. These imply that females late in the flight period had lower residual reproductive values than those flying early, partly because of ageing, partly because they emerged later, for example, with a poor initial condition. Furthermore, females with narrower thoraces had a higher risk to disappear at a given time point from the population than wider females. Conclusions on the time period of a state cannot be directly drawn from the multistate model, that is, higher risk to disappear at a given time point is not equivalent to a shorter presence in the population; the latter depends on the entire event history. However, in our model, no transitions were detected from the state disappear (Figure 5). This supports the hypothesis that females with narrower thoraces disappear sooner than wider females. Disappearance may be due to emigration, undetectability (lower activity), or death. Also, females entering the population late in the flight period had shorter observation periods (rho = −0.12, *p* < .009, *n* = 492 all years combined, Spearman's rank correlation test; Figure S10). We assume that observation periods are associated with lifespan, thus short‐living small females had less time to lay eggs. Males of the *Ephestia kuehniella* invested more in larger and younger than in smaller and older females (Xu & Wang, 2009). In Clouded Apollos, males later in the flight period were probably more constrained on investing in CAPs for the same reasons as female quality deterioration, as well as body reserve depletion (Stjernholm & Karlsson, 2000). Depletion due to resources used for CAP production over consecutive matings of an individual was found in other papilionid species in laboratory conditions (Matsumoto, 1987; Niihara & Watanabe, 2009; Orr, 2002). All these dynamic changes are likely to influence operational sex ratios, therefore, the level of competition for mating in males and the actual investment decisions, and ultimately the CAP‐types produced.

### 4.5 Constrained polyandry

The females of this Clouded Apollo population are potentially polyandrous, regardless of their willingness for multiple mating or disability to resist males that force copulations. Since most females receive shields, probably at their first mating, and shields most often persist over life preventing future matings, shields are likely an important component of reproductive load, the cost imposed on reproductive success for both sexes by male–male competition for mating (Holland & Rice, 1999; Okada et al., 2021) and severely constrain polyandry. In consequence, most females, probably especially the larger, younger and at an early phase of the flight period, are (i) deprived of postcopulatory female choice and (ii) their progeny have reduced genetic diversity compared to multiply mated females. Postcopulatory female choice was found to enhance reproductive success in a wide range of taxa including insects (Firman et al., 2017). Depriving females of this opportunity indicates a high level of intersexual conflict and an evolutionary phase when females seem to be losing to males in the arms race. However, this would be mitigated if Clouded Apollo females were able to reject poor‐quality males as in the papilionid butterfly *Cressida cressida* (Orr, 1999). Multiple mating may also increase female fitness through enhanced genetic variability in their offspring compared to monogamous females through bet‐hedging, that is, in a fluctuating environment, at least some of the offspring would likely to survive (Jennions & Petrie, 2000). This in turn might severely affect populations transitioning from relatively stable to unstable environments such as caused by climate change‐driven unpredictable weather conditions or habitat change by extensive forest management.

## 5 CONCLUSION

This multi‐year study is unique in investigating male investment into mate‐guarding, female remating and its association with female size and adult sex ratio in a natural insect population. In Clouded Apollos, shields were the most frequent mate‐guarding devices and were more persistent than the smaller stopples or filaments, that is, small CAPs. Presumably, due to their larger size, shields are much more costly to produce and fix on the female than small CAPs. The net benefits from shields compared to small CAPs seemed to decline over the progress of the flight period. We propose that the final outcome of CAP production, that is, the CAP‐type produced and the size of the CAP depends on the relative quality of the mates at an actual mating attempt, as well as operational sex ratios and the progress of the flight period. Future experimental studies should test these assumptions to discern the role of body size, reserve depletion, ageing and the expected residual lifespan of the parties, as well as operational sex ratio. Investigating the capacity of females to control mating duration, and in consequence, CAP‐type and size would also be essential to understand the dynamics of CAP production. ‘High quality’ females were more deprived of postcopulatory female choice and genetic variance in their offspring may be reduced compared to the ‘low quality’, therefore, more polyandrous females. Addressing the potential costs and benefits associated with this pattern would further enhance our understanding of the evolution of mating systems with CAP production.

## AUTHOR CONTRIBUTIONS

**Ádám Gór:** Conceptualization (supporting); data curation (equal); formal analysis (lead); investigation (supporting); methodology (supporting); visualization (equal); writing – original draft (lead); writing – review and editing (lead). **Kata Pásztor:** Data curation (equal); investigation (supporting); writing – review and editing (supporting). **Zsolt Lang:** Formal analysis (supporting); supervision (equal); writing – original draft (supporting); writing – review and editing (supporting). **Viktor Szigeti:** Conceptualization (supporting); investigation (supporting); methodology (supporting); writing – review and editing (supporting). **Flóra Vajna:** Investigation (supporting); writing – review and editing (supporting). **János Kis:** Conceptualization (lead); investigation (lead); methodology (lead); project administration (lead); supervision (equal); visualization (equal); writing – original draft (supporting); writing – review and editing (supporting).

## CONFLICT OF INTEREST STATEMENT

The authors declare no conflicts of interest.

## Supporting information


Appendix S1

## Data Availability

The data underlying this article are available in the Zenodo Digital Repository, at https://doi.org/10.5281/zenodo.7962921.
