# Supplementary material for: Mate‐guarding success depends on male investment in a butterfly
Source: Ecol Evol. 2023 Sep 17;13(9):e10533. doi: 10.1002/ece3.10533 (PMC10505759; doi:10.1002/ece3.10533)
Supplement: Supplementary file 1 — Appendix S1 [file ECE3-13-e10533-s001.pdf]

Supplemental Information for:

Mate-guarding success depends on male investment in a butterfly

Ádám Gór, Zsolt Lang, Kata Pásztor, Viktor Szigeti, Flóra Vajna, János Kis

Table S1. Annual variation in the lengths of the flight periods, the total number of individuals, and the number of Clouded Apollo females captured.

Year	First day	Last day	Duration (days)	$N_{individuals}$ (total=1108)	$N_{females}$ (total=492)
**2015**	2015-04-26	2015-05-30	35	176	84
**2016**	2016-04-22	2016-06-03	43	194	88
**2017**	2017-04-25	2017-05-29	35	189	87
**2018**	2018-04-29	2018-05-24	26	271	116
**2019**	2019-04-21	2019-06-04	45	203	83
**2020**	2020-04-21	2020-05-21	31	75	34

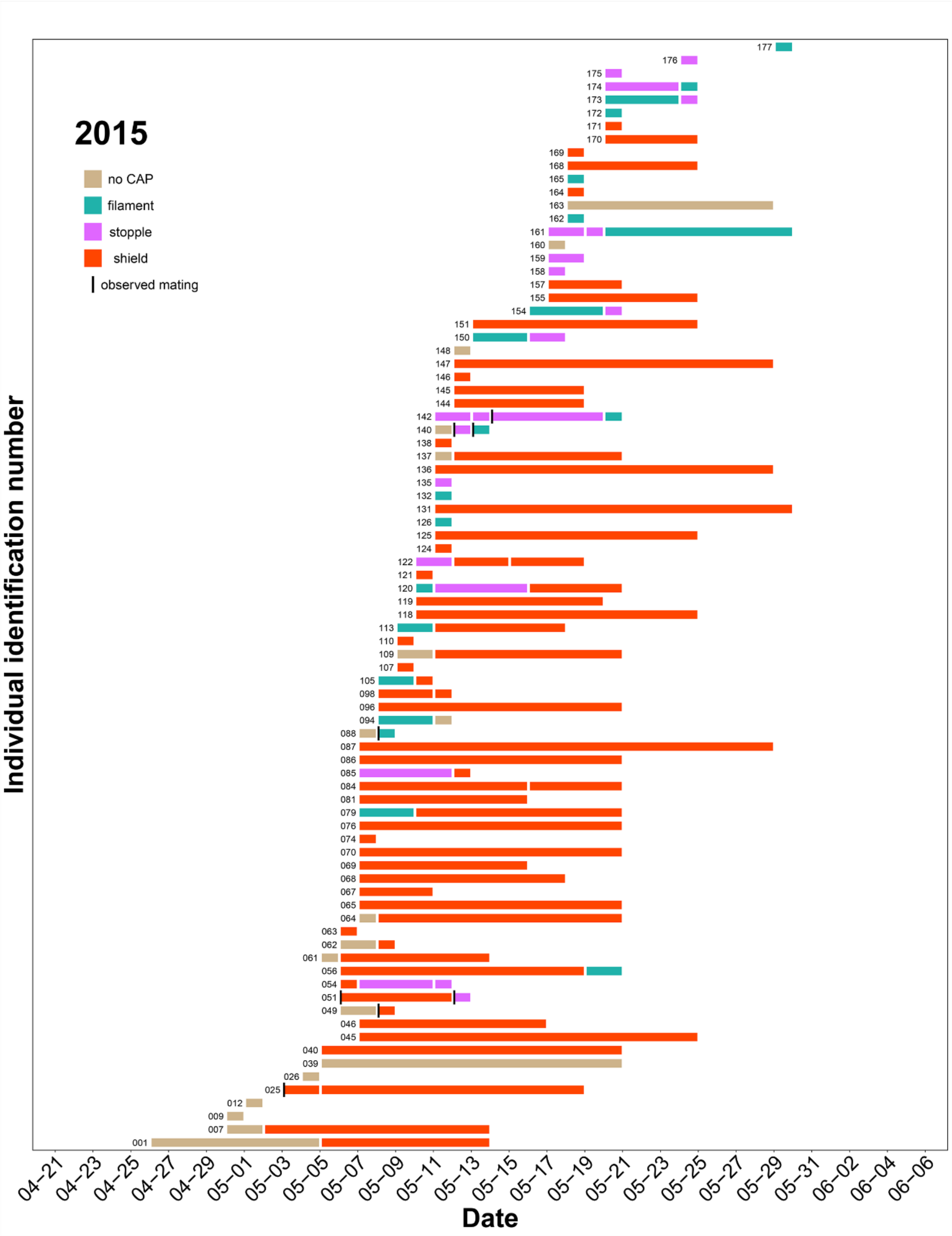

Figure S1. Copulatory opening APpendix (CAP) event histories in 2015; actual observations on Clouded Apollo females. Each horizontal row (ordered by the annual individual identification numbers from bottom to top) represents the history of a female butterfly and row length shows the individuals' observation period. Colour-coded segments indicate the different CAP-types. Vertical black bars show the observed matings.

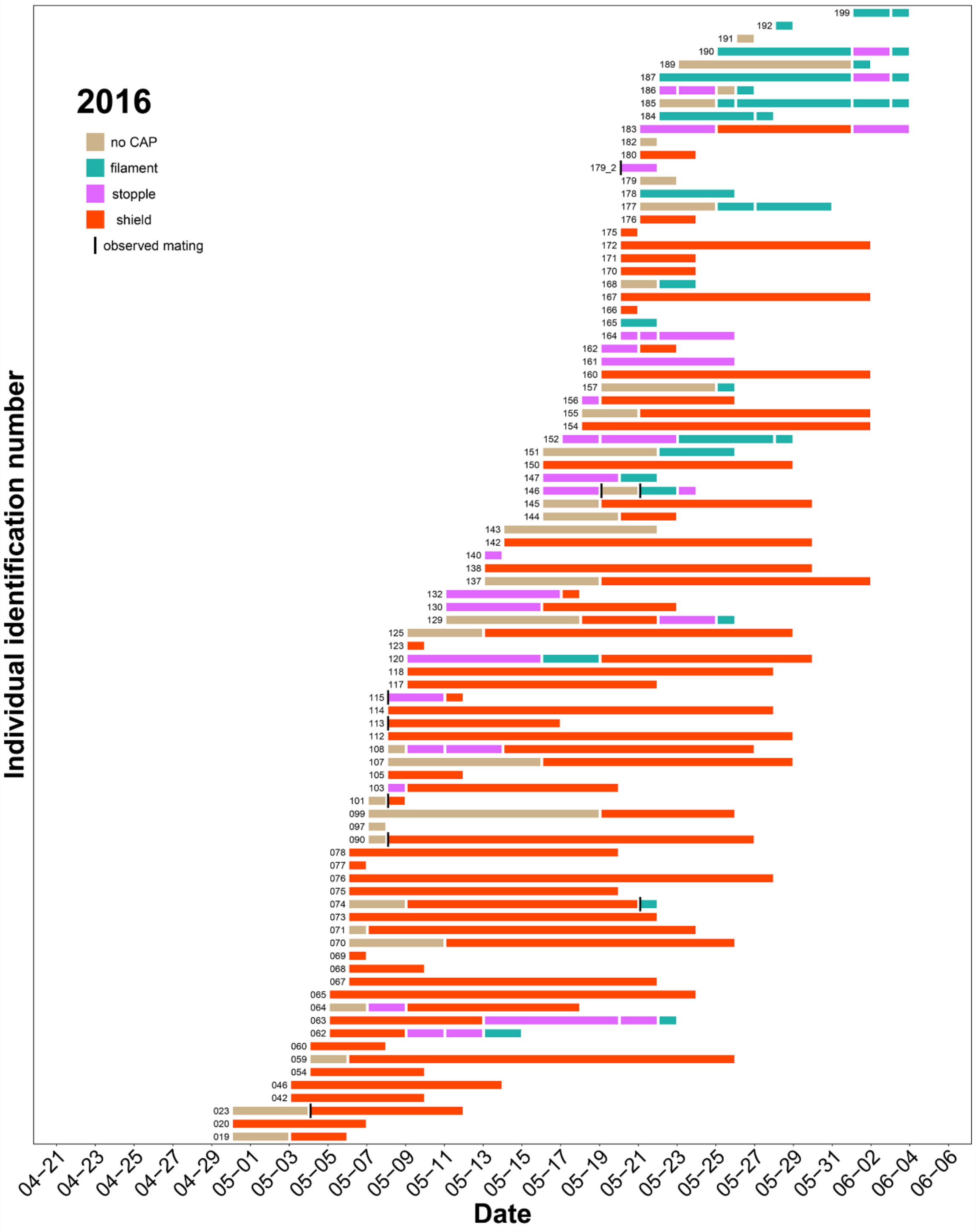

Figure S2. Copulatory opening APpendix (CAP) event histories in 2016; actual observations on Clouded Apollo females. Each horizontal row (ordered by the annual individual identification numbers from bottom to top) represents the history of a female butterfly and row length shows the individuals' observation period. Colour-coded segments indicate the different CAP-types. Vertical black bars show the observed matings.

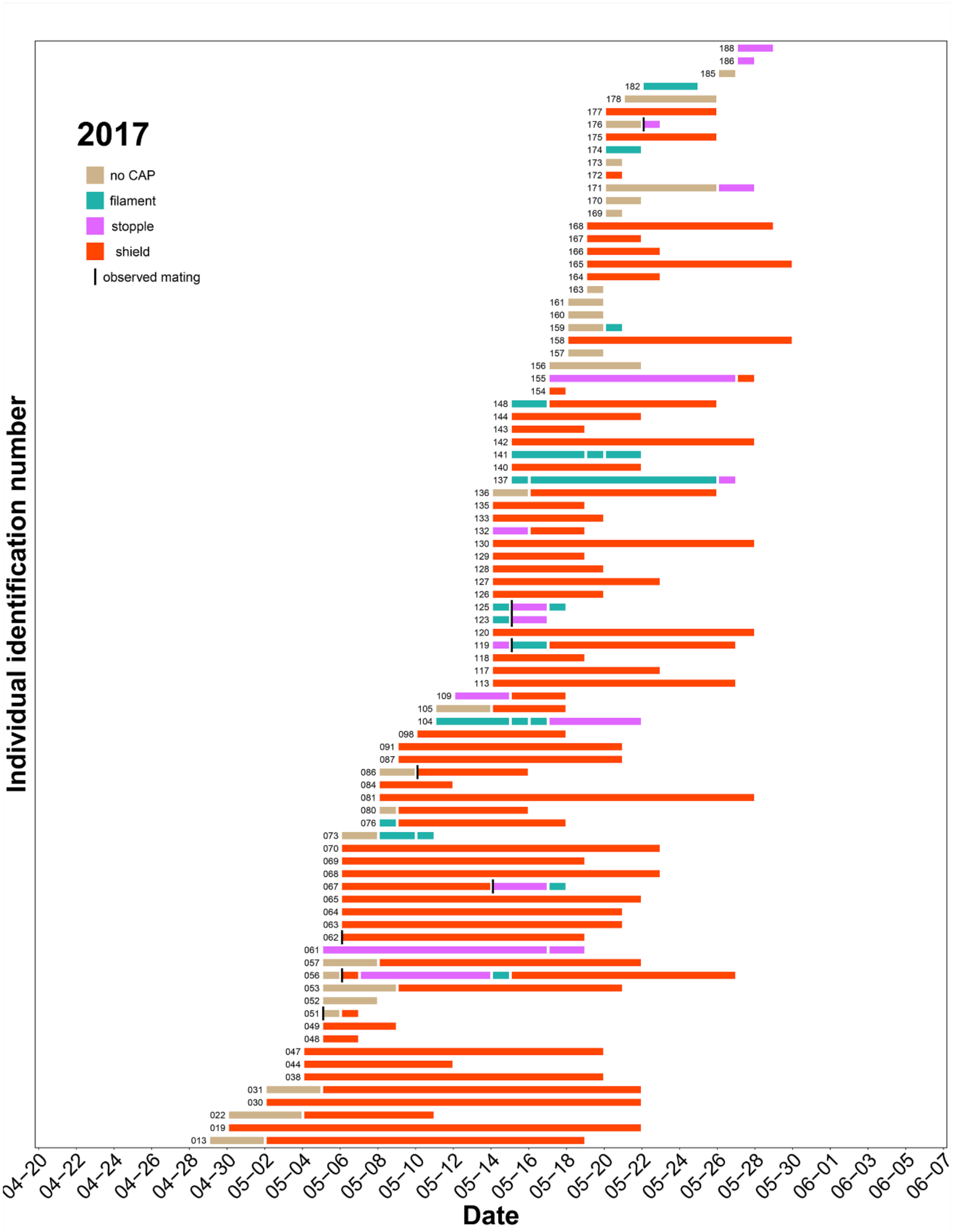

Figure S3. Copulatory opening APpendix (CAP) event histories in 2017; actual observations on Clouded Apollo females. Each horizontal row (ordered by the annual individual identification numbers from bottom to top) represents the history of a female butterfly and row length shows the individuals' observation period. Colour-coded segments indicate the different CAP-types. Vertical black bars show the observed matings.

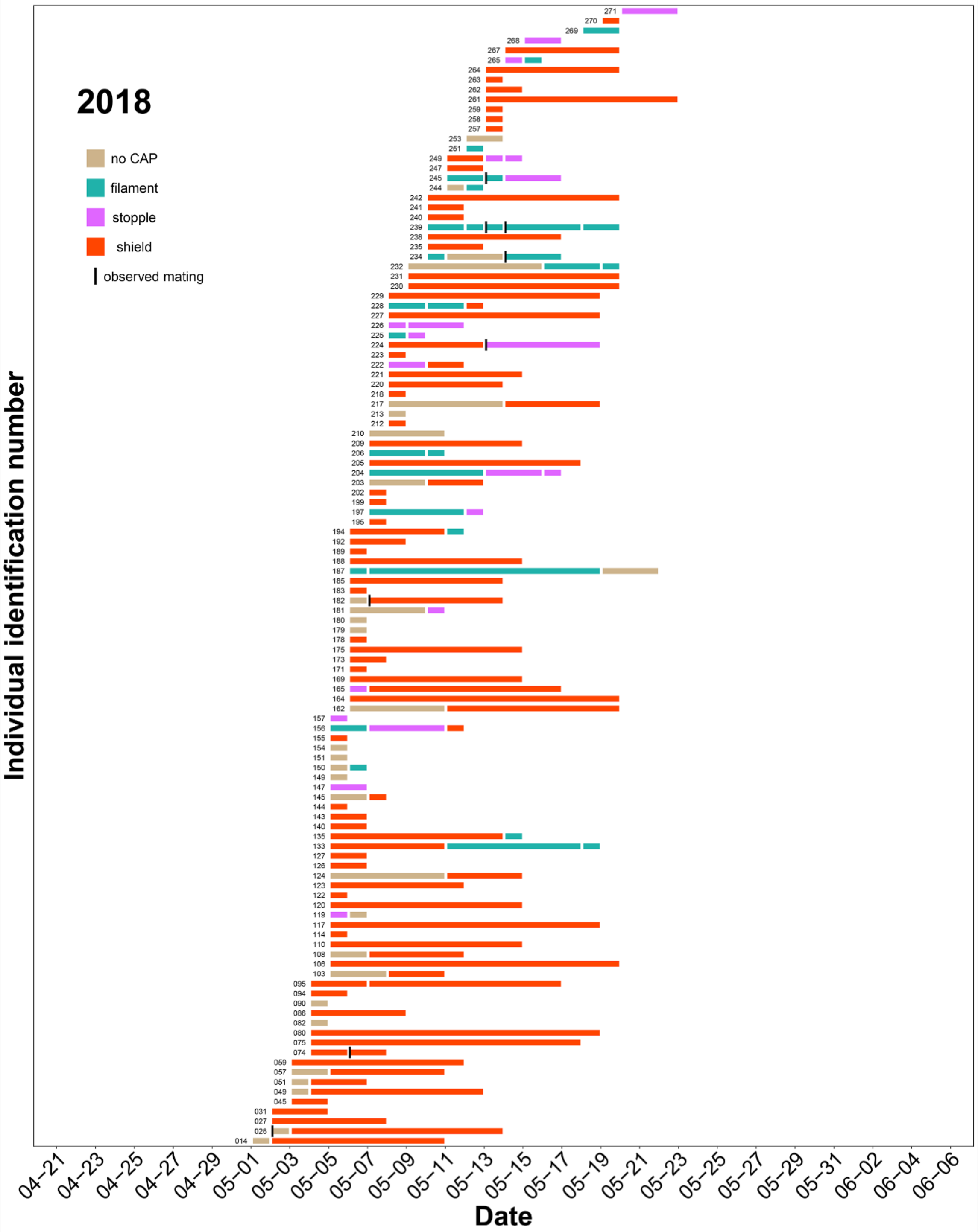

Figure S4. Copulatory opening APpendix (CAP) event histories in 2018; actual observations on Clouded Apollo females. Each horizontal row (ordered by the annual individual identification numbers from bottom to top) represents the history of a female butterfly and row length shows the individuals' observation period. Colour-coded segments indicate the different CAP-types. Vertical black bars show the observed matings.

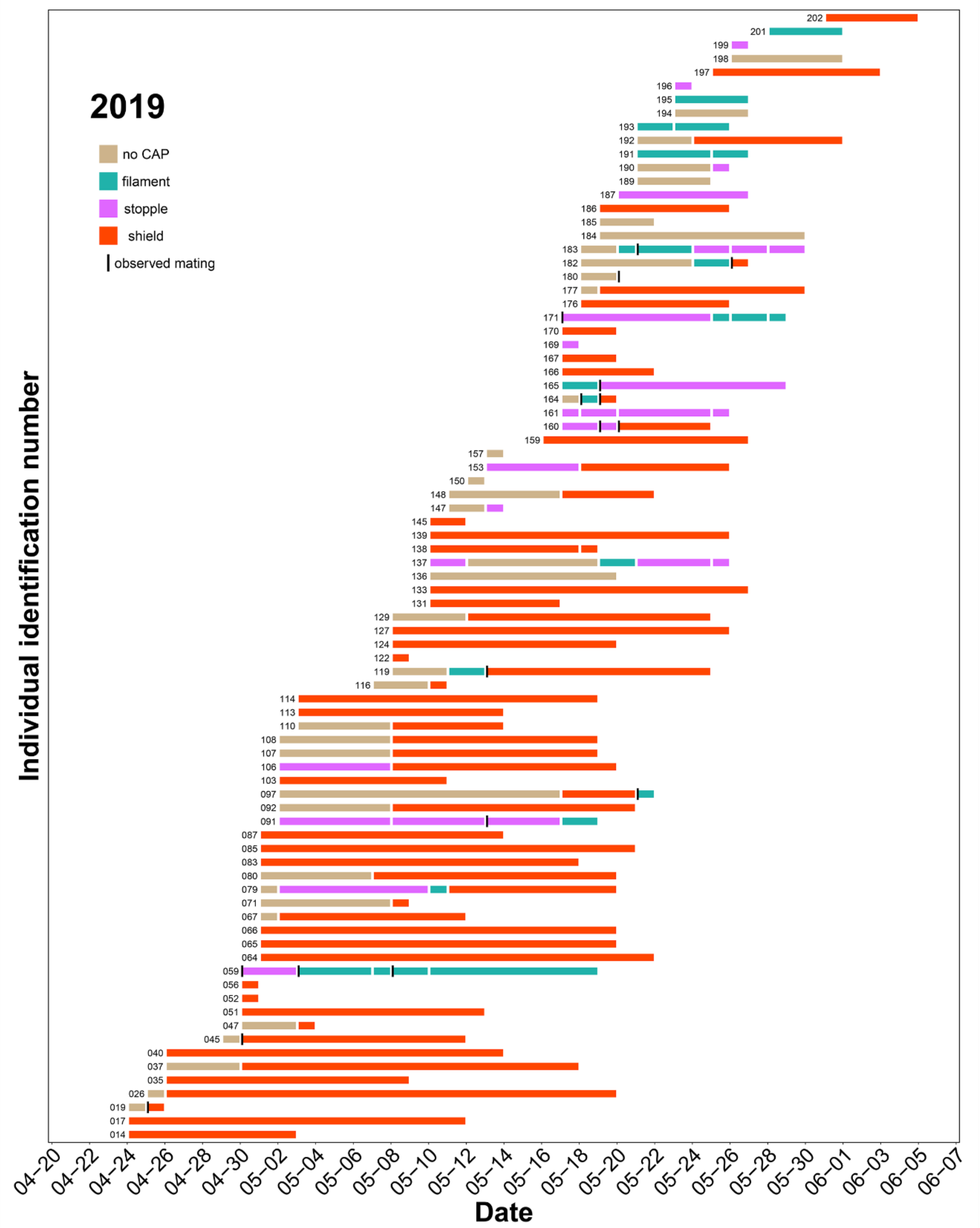

Figure S5. Copulatory opening APpendix (CAP) event histories in 2019; actual observations on Clouded Apollo females. Each horizontal row (ordered by the annual individual identification numbers from bottom to top) represents the history of a female butterfly and row length shows the individuals' observation period. Colour-coded segments indicate the different CAP-types. Vertical black bars show the observed matings.

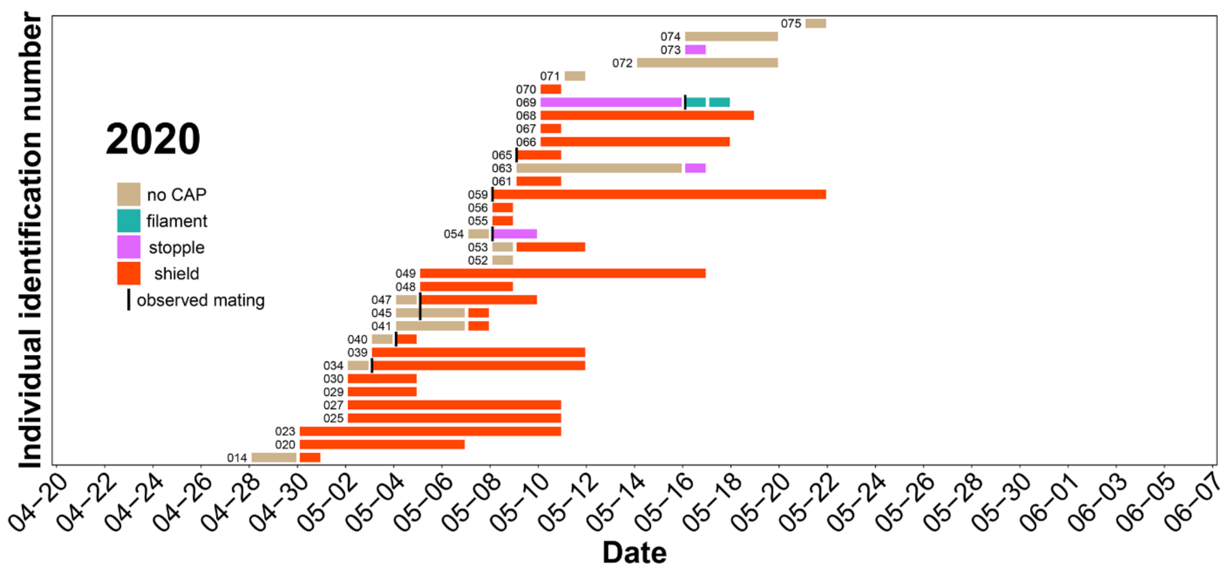

Figure S6. Copulatory opening APpendix (CAP) event histories in 2020; actual observations on Clouded Apollo females. Each horizontal row (ordered by the annual individual identification numbers from bottom to top) represents the history of a female butterfly and row length shows the individuals' observation period. Colour-coded segments indicate the different CAP-types. Vertical black bars show the observed matings.

Table S2. Frequencies of the different transitions over six years in Clouded Apollo females, used in the semiparametric multistate survival model. The table shows the annual and the total number of transitions with the number of females gone through a specific transition in parentheses. Although most females were first caught already bearing a Copulatory opening APendix (CAP), we assumed that all started their lives as no CAP. Transitions to disappeared show the last observed state of any female, thus all these states and females overlap with those of other transitions, since ultimately, all females disappeared by the end of the flight period.

Transition / year	2015	2016	2017	2018	2019	2020	Total	Ref. to Figure 5.
**no CAP → small CAP**	26 (26)	33 (31)	20 (20)	26 (25)	25 (24)	4 (4)	134 (130)	B
**no CAP → shield**	51 (51)	52 (52)	56 (56)	81 (81)	50 (50)	25 (25)	315 (315)	A
**no CAP → disappeared**	8 (8)	5 (5)	11 (11)	12 (12)	9 (9)	5 (5)	50 (50)	I
**small CAP → no CAP**	1 (1)	2 (2)	0	3 (3)	1 (1)	0	7 (7)	D
**small CAP → small CAP**	12 (9)	25 (16)	15 (10)	19 (14)	24 (11)	4 (1)	99 (61)	F
**small CAP → shield**	6 (6)	10 (10)	7 (7)	4 (4)	7 (7)	0	34 (34)	H
**small CAP → disappeared**	22 (22)	26 (26)	15 (15)	24 (24)	18 (18)	4 (4)	109 (109)	K
**shield → small CAP**	3 (3)	5 (5)	2 (2)	5 (5)	1 (1)	0	16 (16)	G
**shield → shield**	4 (4)	0	0	2 (2)	1 (1)	0	7 (7)	E
**shield → disappeared**	54 (54)	57 (57)	61 (61)	80 (80)	56 (56)	25 (25)	333 (333)	J
**Total**	187 (84)	215 (88)	187 (87)	256 (116)	192 (83)	65 (34)	1102 (492)	-

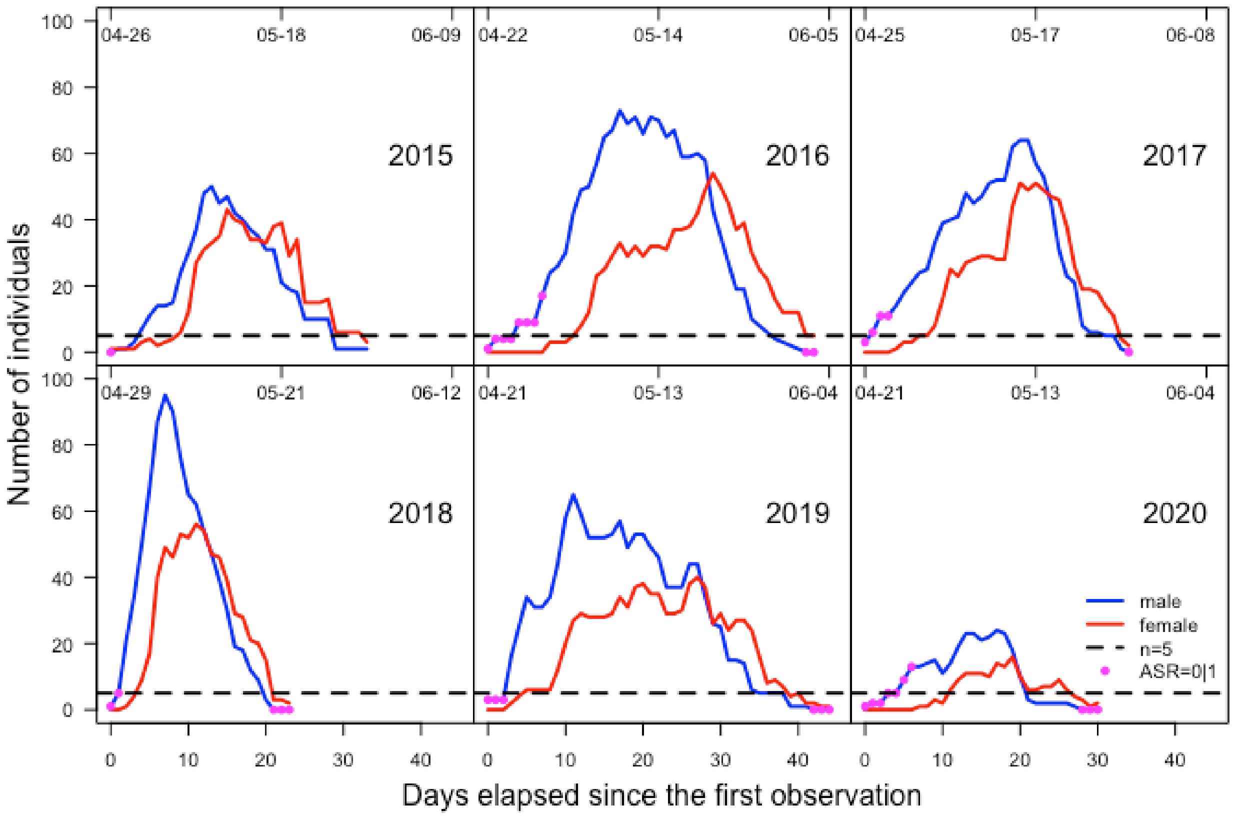

Figure S7. The daily number of Clouded Apollo males (blue lines) and females (red lines) during the flight period from 2015 to 2020. The top horizontal axes show dates; note the differences across years. Black dashed lines show the threshold where the number of individuals equals 5. Magenta dots mark days where adult sex ratios (ASR) were equal either to zero or one.

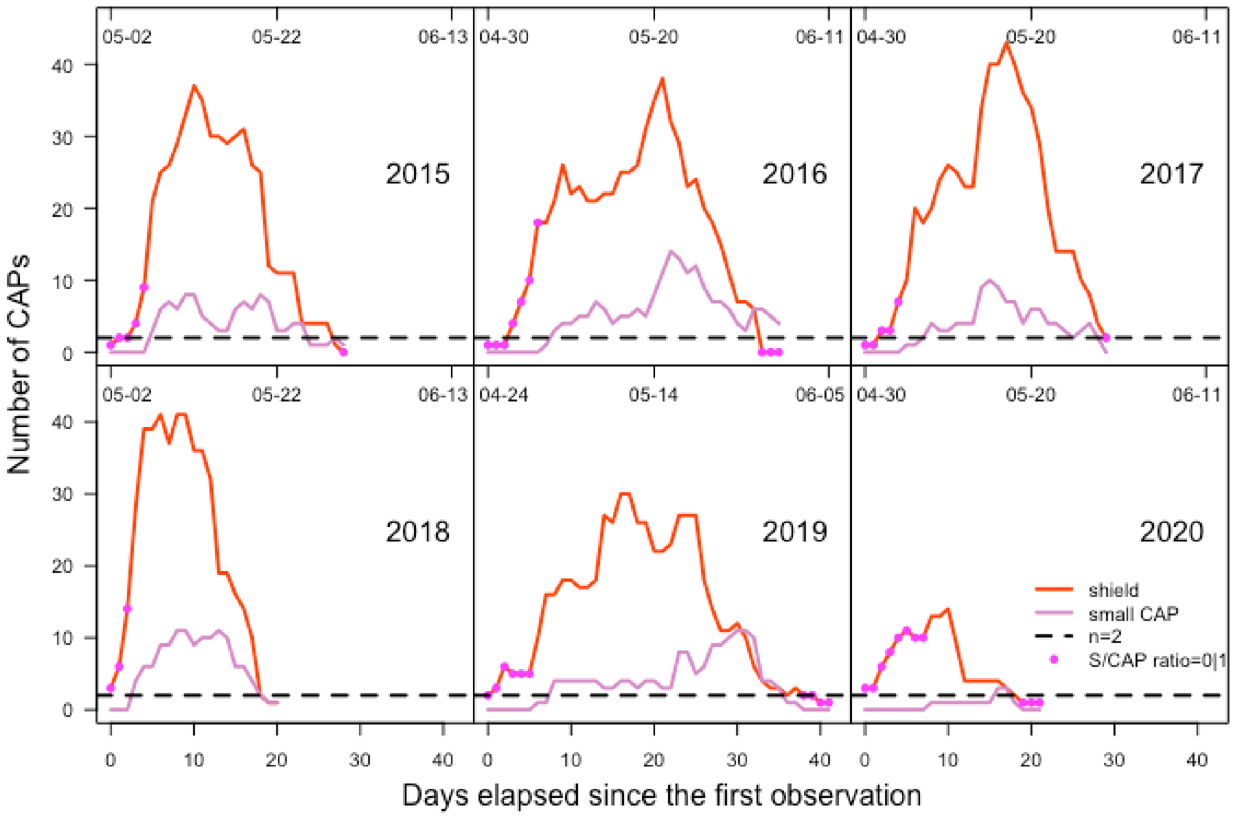

Figure S8. The daily number of Copulatory opening APpendix (CAP) types: shields (orange lines) and small CAPs (purple lines) during the flight period from 2015 to 2020 in Clouded Apollos. The top horizontal axes show dates; note the differences across years. Black dashed lines show the threshold where the number of CAPs equals 2. Magenta dots mark days where shield to CAP ratios were equal either to zero or one.

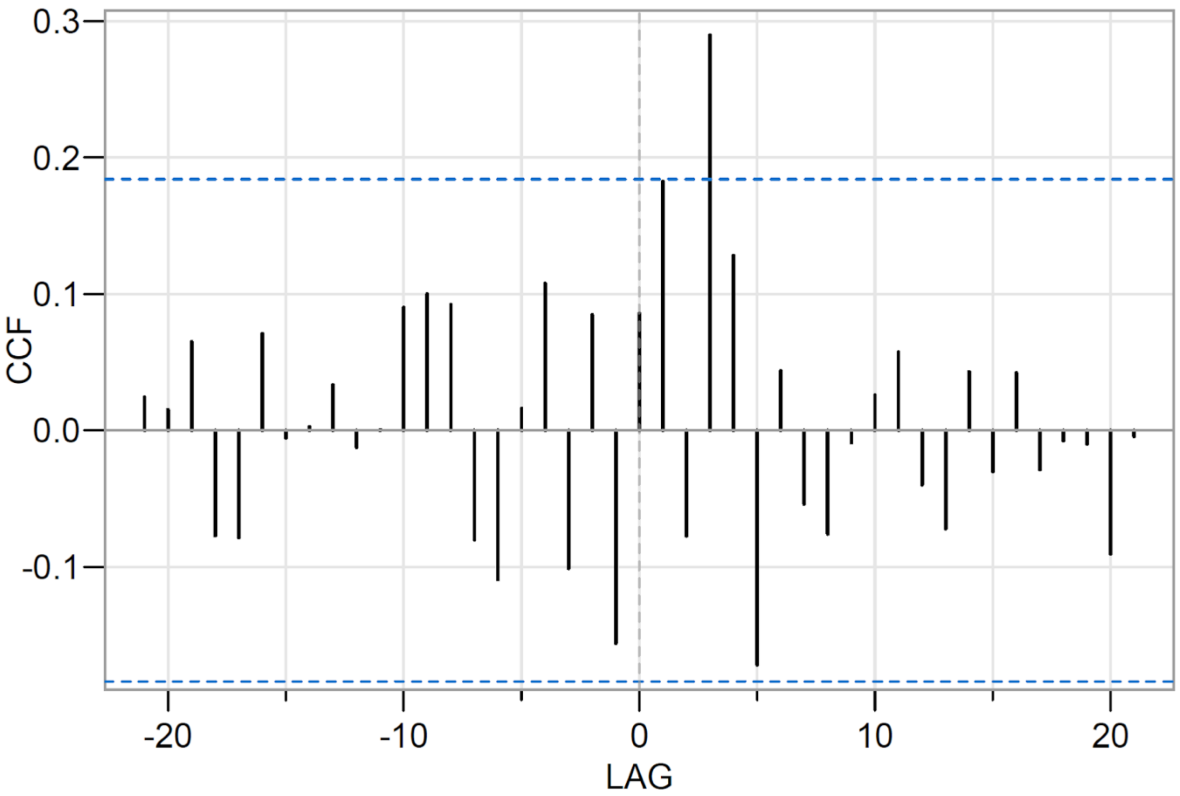

Figure S9. Cross-correlation function (CCF) computed between the time series of the adult sex ratios and shield to CAP ratios in Clouded Apollos. The time lag is measured in days. The blue dashed line is the 95% confidence interval for the correlation coefficients.

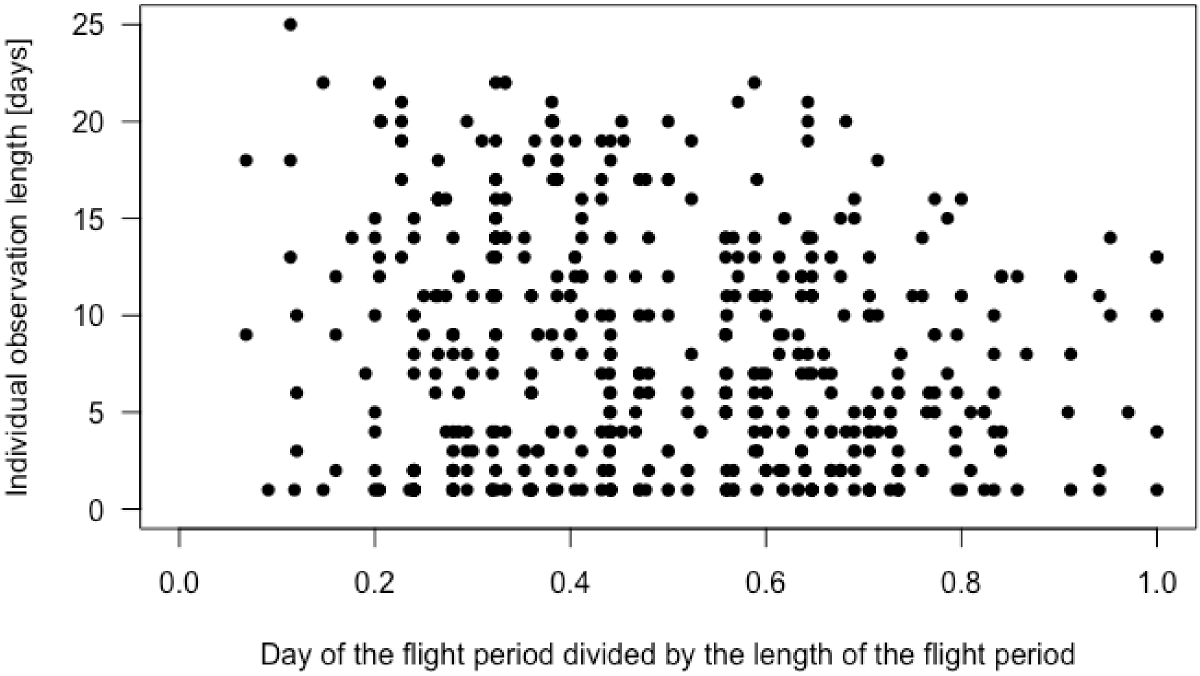

Figure S10. Individual observation length over the flight period between 2015 and 2020 in Clouded Apollos.

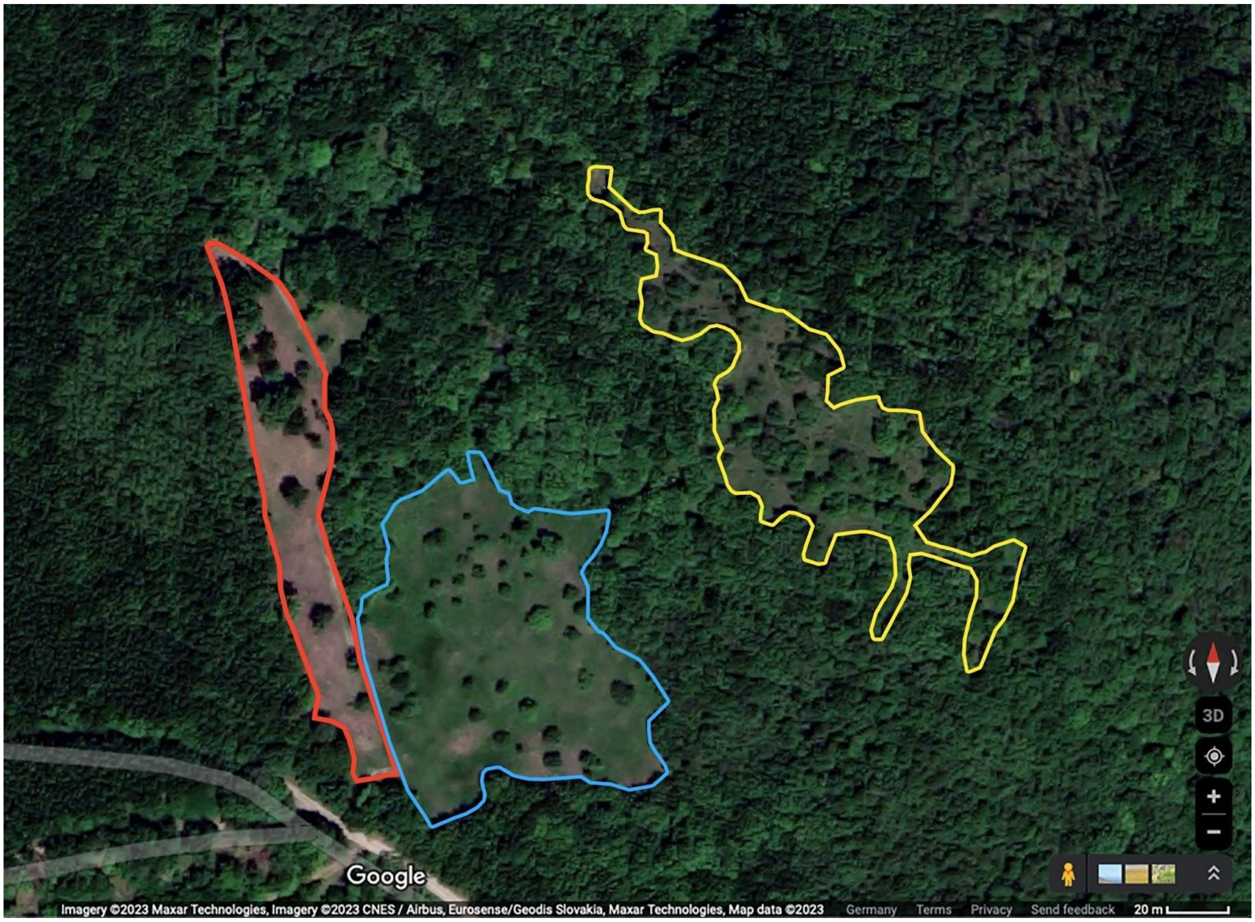

Figure S11. Aerial photograph on the study site and its surroundings (obtained via © Google Maps, 2023). Yellow denotes the area where most Clouded Apollos were caught and observed, a hilltop and a SW slope. Blue denotes a seemingly suitable habitat at a lower elevation, where Clouded Apollos were only occasionally caught or observed. Red denotes a fresh clearing (2020) without suitable forbs for Clouded Apollos.

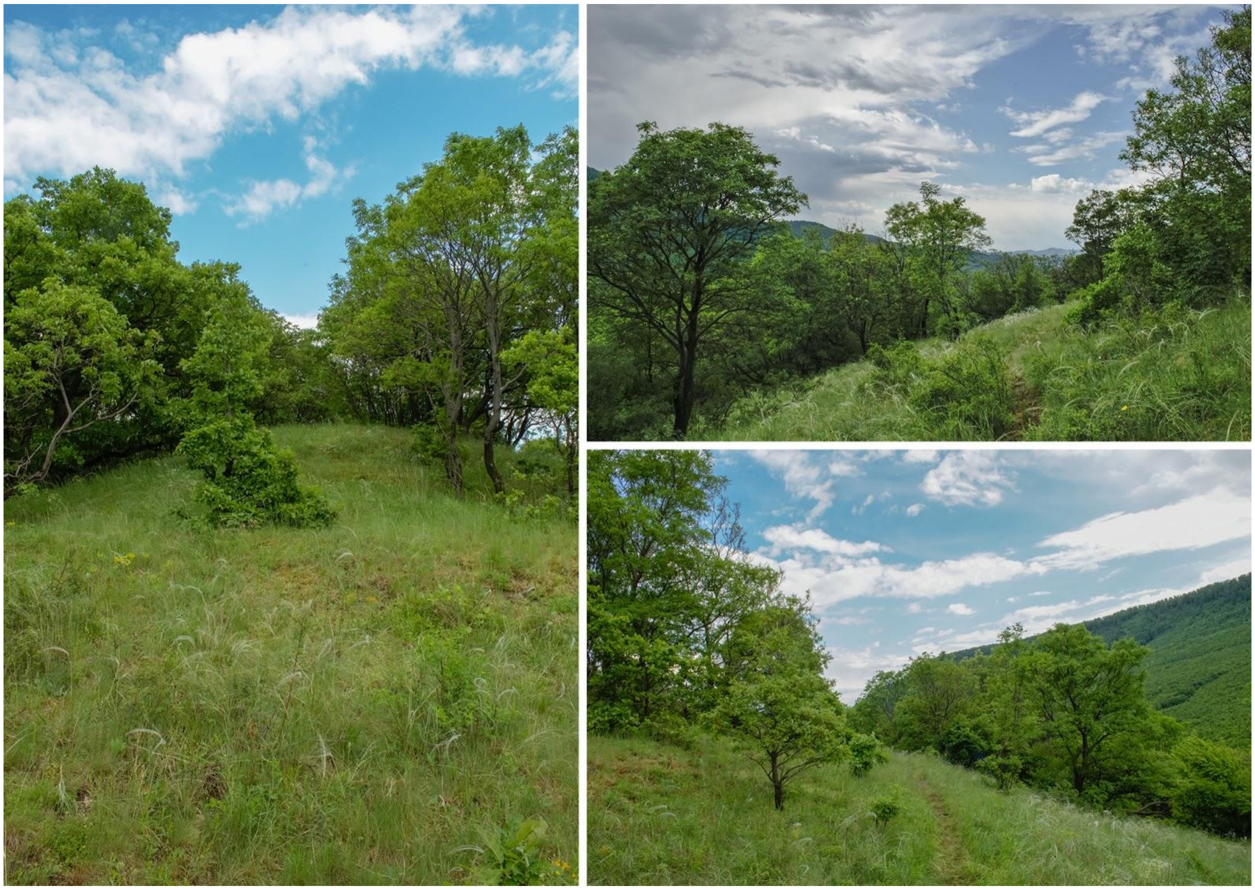

Figure S12. Habitat photographs of the study site. Note the patchy character of the open meadow, scattered with small groups of trees and shrubs. Photos were taken by JK.
